# Differential regulation of germ line apoptosis and germ cell differentiation by CPEB family members in *C*. *elegans*

**DOI:** 10.1371/journal.pone.0182270

**Published:** 2017-07-31

**Authors:** Kapil Dev Singh, Xue Zheng, Stuart Milstein, Martin Keller, Bernd Roschitzki, Jonas Grossmann, Michael O. Hengartner

**Affiliations:** 1 Institute of Molecular Life Sciences, University of Zurich, Zurich, Switzerland; 2 PhD Program in Molecular Life Science, University of Zurich and ETH Zurich, Zurich, Switzerland; 3 Cold Spring Harbor Laboratory, Cold Spring Harbor, New York, United States of America; 4 Functional Genomics Center Zurich, University of Zurich and ETH Zurich, Zurich, Switzerland; East Carolina University, UNITED STATES

## Abstract

Cytoplasmic polyadenylation element binding (CPEB) proteins are evolutionary conserved RNA-binding proteins that control mRNA polyadenylation and translation. Orthologs in humans and other vertebrates are mainly involved in oogenesis. This is also the case for the *C*. *elegans* CPEB family member CPB-3, whereas two further CPEB proteins (CPB-1 and FOG-1) are involved in spermatogenesis. Here we describe the characterisation of a new missense allele of *cpb-3* and show that loss of *cpb-3* function leads to an increase in physiological germ cell death. To better understand the interaction and effect of *C*. *elegans* CPEB proteins on processes such as physiological apoptosis, germ cell differentiation, and regulation of gene expression, we characterised changes in the transcriptome and proteome of *C*. *elegans* CPEB mutants. Our results show that, despite their sequence similarities CPEB family members tend to have distinct overall effects on gene expression (both at the transcript and protein levels). This observation is consistent with the distinct phenotypes observed in the various CPEB family mutants.

## Introduction

The central dogma of molecular biology states that genetic information generally flows from DNA through RNA to proteins [1]. In eukaryotic organisms this information transfer is highly regulated at each step of the process, including at the level of post-transcriptional regulation of mRNAs (such as mRNA splicing, transport, localization, stability, and translational activation), which often involves RNA-binding proteins (RBPs) and microRNAs (miRNAs) [2,3].

Cytoplasmic polyadenylation element binding (CPEB) proteins are RBPs that bind to cytoplasmic polyadenylation elements (CPEs), in the 3’-UTR region of mRNAs [4,5]. CPEB proteins act within a large ribonucleoprotein (RNP) complex and are mainly involved in the regulation of polyadenylation [4]. They are also indirectly involved in both translational activation and repression as well as in cell differentiation, division, and senescence [6]. CPEB proteins are characterized by two RNA recognition motifs (RRMs) and a zinc finger (ZnF)-like cysteine-histidine repeat region (C/H domain; composed of C_4_ and C_2_H_2_ motifs) [7]. Both RRMs and the C/H domain are essential for specific RNA binding [8]. CPEB proteins are evolutionarily conserved and present in all metazoans, from invertebrates to humans. Vertebrates often contains four paralogs, whereas invertebrates have been found to contain only one or two [9]. *Caenorhabditis elegans* is an exception as it contains four CPEB proteins (CPB-1, CPB-2, CBP-3, and FOG-1) [7,10]. All nematode species analysed had four CPEB family members, suggesting an expansion either very early in the nematode lineage or slightly prior to their separation from other lineages [10].

CPEB proteins are involved in oogenesis in many organisms, including flies [11], zebrafish [12], frogs [4,13], mice [14], and humans [15]. CPEB proteins also play important roles in memory formation and neuronal function [16,17]. In *C*. *elegans* the closest ortholog of vertebrate CPEB proteins is CPB-3 [18] (S1 Fig). Like its vertebrate relatives, CPB-3 is involved in the regulation of oogenesis, promoting entry into and progression of oocytes through meiosis [19]. Additionally, loss of CPB-3 results in increased level of physiological apoptosis in germ cells [20,21], but the underlying genetic mechanisms are not yet understood.

In contrast to CPB-3, CPB-1 and FOG-1 are both required for spermatogenesis, with FOG-1 participating in sperm cell fate determination and CPB-1 contributing to spermatocyte differentiation [7,22]. Little is known about the function of CPB-2, except that its transcript is highly expressed during spermatogenesis [7,23], suggesting that *cpb-2*, like *cpb-1* and *fog-1*, might also be involved in this process.

Here we describe the isolation and characterisation of a new missense allele of *cpb-3*. We show that this mutation leads to defects in oogenesis and increased levels of p53-independent apoptosis in the adult hermaphrodite germ line. To better understand the interaction and the effect of *C*. *elegans* CPEB proteins on processes such as physiological apoptosis, germ cell differentiation, and regulation of gene expression, we characterised changes in the transcriptome and proteome of *fog-1*, *cpb-2*, and *cpb-3* mutants. Consistent with the distinct phenotypes caused by loss of the various family mutants, we find that CPEB family members tend to have distinct overall effects on gene expression (both at the transcript and protein levels).

## Results and discussion

### Characterisation of the allele *op234*

The *op234* allele was originally isolated in a forward genetic screen designed to identify novel genes that control germ line apoptosis (gla). *op234* was originally assigned to a novel gene name “*gla-1*” as it did not map to any previously known cell death gene [24].

**Fig 1:**
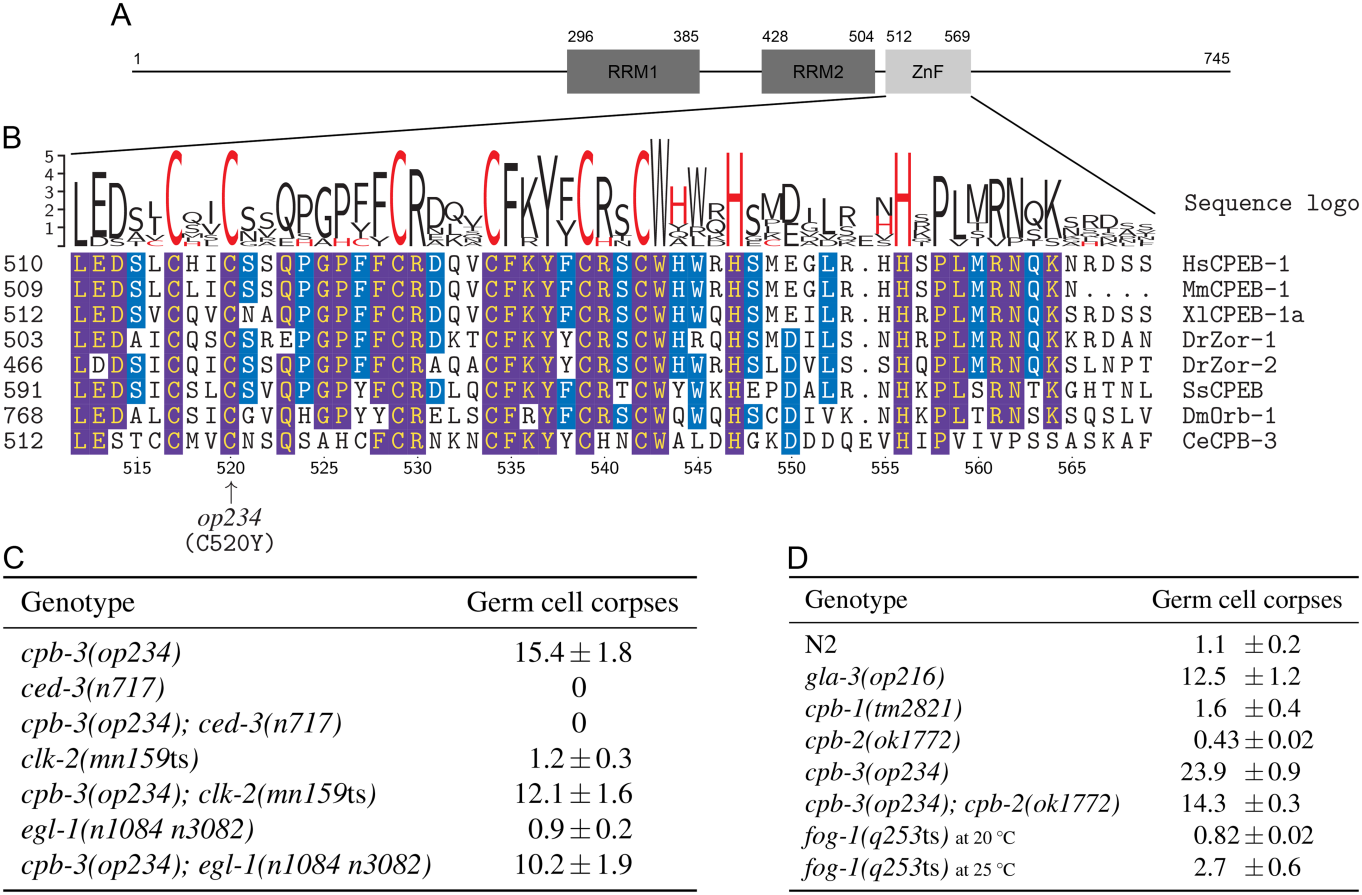
Characterisation of *cpb-3(op234)*. (A) Schematic of the CPB-3 protein, showing the position of its two RRMs and the C/H domain (ZnF). (B) Multiple sequence alignment of the ZnF motifs from clade-I CPEB homologs (see S1 Fig). Frequency corrected sequence logos [25] are shown above the alignment (cysteine and histidine amino acids are in red). Residue numbers at the bottom of the alignment refer to CPB-3; arrow indicates the position of the missense mutation (C520Y) in the *cpb-3(op234)* allele. Identical amino acids are highlighted in blue for more than 50% sequence identity, and in purple for more than 80% sequence identity. (C and D) Loss of *cpb-3*, but not other CPEB family members, leads to increased physiological germ cell death. Data shown are average number of germ cell corpses ± SEM from three (panel C) and two (panel D) independent experiments, scored in animals of the indicated genotypes 30 h (45 h for *cpb-2* due to slow growth) post L4/adult molt, with at least 15 gonads in each experiment. Note: germ cell corpse counting for panel C and D was performed by different investigators, data should thus be compared within each panel but not across panels.

Through classical two- and three-factor as well as SNP mapping, we localised the allele *op234* to a roughly 46 kb region containing 12 genes on chromosome I (see Materials and methods). One of these 12 genes, B0414.5/*cpb-3* gave rise to an increased number of germ cell corpses when inactivated by RNA interference (RNAi). We sequenced the *cpb-3* from wild-type and *op234* mutant worms and detected a G-to-A transition at position 1559 of the spliced gene (position 1724 of the unspliced gene). This transition leads to a missense mutation in the CPB-3 protein, resulting in a non-conservative substitution of an invariant cysteine to tyrosine (C520Y) within the conserved C/H domain (Fig 1A and 1B), which likely leads to a loss of function of this domain. As the molecular nature of the gene affected by *op234* is now known, we will henceforth refer to this allele as *cpb-3(op234)* instead of *gla-1(op234)*.

To determine the epistatic relationship between *cpb-3* and other apoptotic genes, we generated double mutants between *cpb-3(op234)* and the strong loss-of-function mutation *ced-3(n717)*, which lacks the major *C*. *elegans* caspase and is characterised by a complete loss of apoptosis [26]. Virtually no germ cell death was observed in *cpb-3(op234); ced-3(n717)* animals, demonstrating that *op234*-induced cell death is apoptotic in nature and that *cpb-3* functions up stream of the core apoptotic genes (Fig 1C). Since germ line apoptosis can be induced by both physiological signals and through various stresses, including DNA damage [27], we also performed an epistatic analysis with *clk-2* and *egl-1* mutations. *clk-2(mn159*ts*)* animals are characterised by a complete loss of DNA damage-induced apoptosis, but have normal levels of somatic and physiological germ line apoptosis [28], whereas *egl-1(n1084 n3082)* worms are defective in both DNA damage-induced and somatic apoptosis but show normal physiological germ line apoptosis [29,30]. We found that both *clk-2* and *egl-1* failed to suppress the high germ line apoptosis of *cpb-3* (Fig 1C). Increased cell corpse number could in principle result either from an increase in the number of cells that die or in a decrease in the clearance efficiency of dead cells, which would lead to their accumulation. Based on our prior results [24], we can clearly exclude the latter possibility. Taken together, these observations thus support our previous, RNAi-based conclusion that loss of *cpb-3* function results in increased physiological apoptosis in germ cells [21].

**Fig 2:**
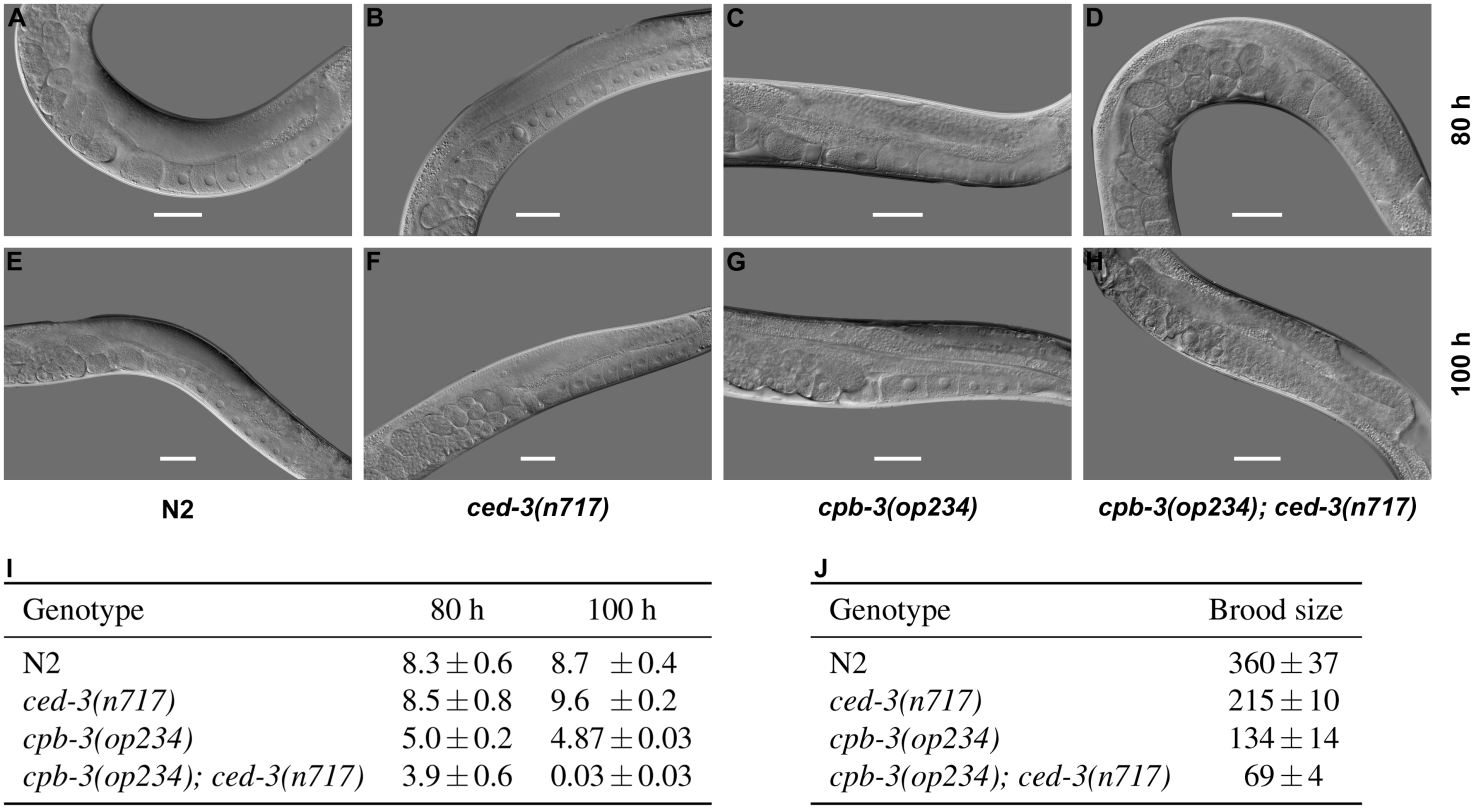
*cpb-3(op234)* animals have oogenic defects. DIC microscopy images of adult hermaphrodite worm gonads at 80 h (A-D) and 100 h (E-H) post hatching. Scale bar: 50 μm. (I) Loss of mature (full size) oocytes in old *cpb-3(op234); ced-3(n717)* double mutants. Data shown are average number of mature oocyte ± SEM from three independent experiments, scored in animals of the indicated genotypes at 80 h and 100 h post hatching, with at least 10 gonads in each experiment. (J) *cpb-3(op234)* animals have a reduced brood size. Data shown are average brood size ± SD from at least 10 worms, scored in animals of the indicated genotypes.

To further understand the role of *cpb-3* in oogenesis and germ cell survival, we compared gonads of *cpb-3(op234)* hermaphrodites with wild type (Fig 2). We observed that in the gonads of older animals, *cpb-3(op234)* mutants contained fewer mature oocytes (full-sized oocytes which span across the whole gonad diameter) than wild type. This phenotype was further enhanced by inactivation of apoptosis in *cpb-3; ced-3* double mutants, leading to a complete loss of mature oocytes in the gonad of older worms (Fig 2H and 2I). Consistent with the above, *cpb-3(op234)* mutants also showed a reduced brood size, which was further decreased in the absence of apoptosis (Fig 2J). These observations show that *cpb-3(op234); ced-3(n717)* animals still have oogenic defects even in the absence of apoptosis, suggesting that *cpb-3* plays a broader role in oogenesis rather than simply regulating germ cell survival. The synergetic defect in oogenesis observed in the *cpb-3(op234); ced-3(n717)* double mutant likely arises from the fact that old *ced-3* mutants also fail to properly generate mature oocytes [30].

We also analysed germ cell apoptosis in the other CPEB mutants. Unlike *cpb-3(op234)* others mutants: *cpb-1(tm2821)*, *cpb-2(ok1772)*, and *fog-1(q253*ts*)* showed little to no change in cell corpse numbers (Fig 1D). Double mutants between *cpb-3(op234)* and *cpb-2(ok1772)* still had high apoptosis levels, albeit slightly less than the *cpb-3* single mutant (Fig 1D). This is consistent with the previous observation of Hasegawa et al., who failed to find any effect of *cpb-2(RNAi)* on the germ line differentiation defects that can also be found in *cpb-3* [19]. This suggests that these two CPEB proteins might have distinct effect on *C*. *elegans* germ line development.

### Proteomics and transcriptomics

To better understand the role of CPB-3 in the regulation of oogenesis and spermatogenesis, we characterised the effect of loss of *cpb-3* function at the transcriptome and proteome levels. In parallel, we also characterised the changes induced by loss of the CPEB family members: *fog-1* (involved in spermatogenesis) and *cpb-2* (function unknown). CPB-1 was not considered due to the absence of a fertile mutant strain.

**Fig 3:**
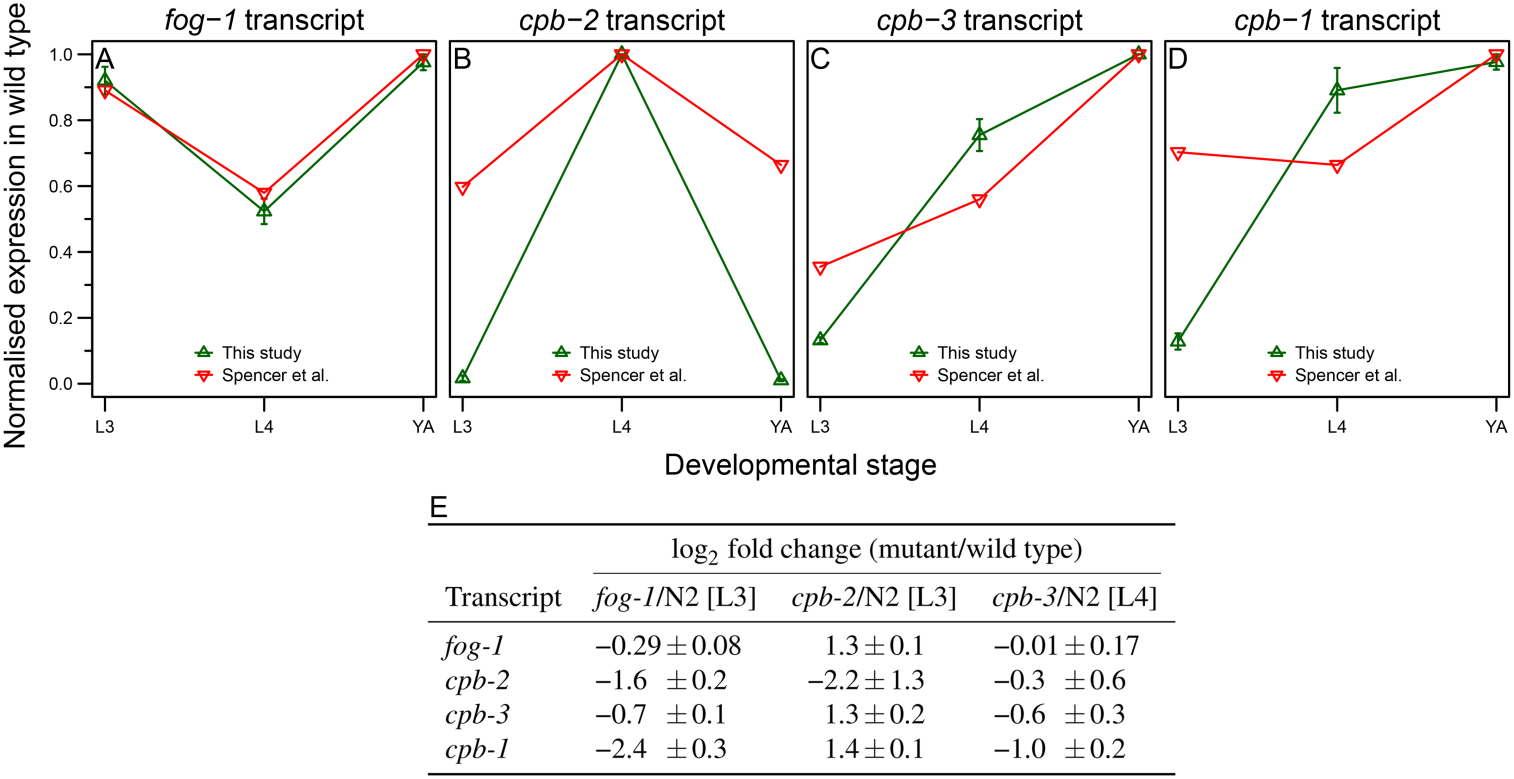
Transcript expression levels of *C*. *elegans* CPEB homologs in wild-type and CPEB mutant animals. (A-D) Transcript expression levels of CPEB homologs in this study show similar trends as in the previously published dataset from Spencer et al. [31]. Normalised expression levels from both studies (normalised counts per million (CPM) in our dataset and normalised mean tiling array expression values in Spencer et al.) are shown for *fog-1* (A), *cpb-2* (B), *cpb-3* (C), and *cpb-1* (D) in wild-type animals at L3, L4, and YA stage. Error bars represent SEM between three biological replicates. The higher sensitivity of RNA-seq compared to tiling array is most likely responsible for the observed differences in CPEB levels between two studies. (E) Change in CPEB transcript levels in the various CPEB mutants relative to wild type. Data shown are average log_2_ scaled fold changes ± SEM. SEM in fold change between three biological replicates was calculated from the respective normalised CPM ratios.

All four *C*. *elegans* CPEB family members are expressed specifically in the germ line, but with distinct temporal expression pattern [31] (Fig 3A–3D). Based on these expression patterns and the known phenotype of *fog-1* and *cpb-3* mutants, we selected larval stage 3 (L3) for *fog-1(q253*ts*)* and *cpb-2(ok1772)* animals, and L4 for *cpb-3(op234)* mutants. Those stages were chosen one stage prior to the full-blown phenotype in order to give us a chance to identify early changes in gene expression that might contribute to the mutant phenotype.

**Fig 4:**
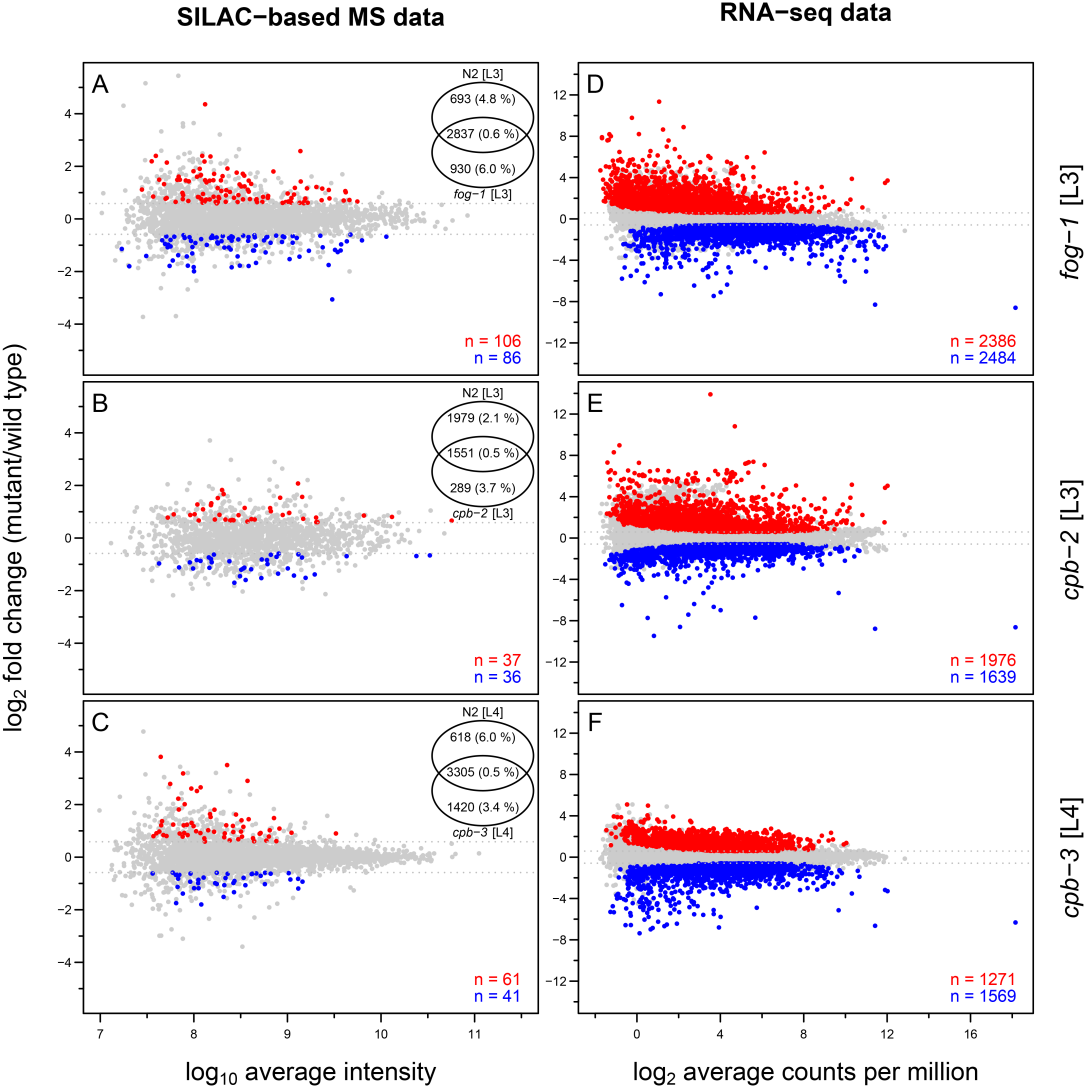
Overview of SILAC-based MS and RNA-seq data for CPEB mutants in *C*. *elegans* relative to wild type. M (log-fold change) versus A (log-average expression) plots for SILAC-based MS data (A-C) and RNA-seq data (D-F). Differentially expressed protein groups (|FC| > 1.5 and *P*-value < 0.05) and transcripts (|FC| > 1.5 and BH-adjusted *P*-value < 0.01) are shown in red for overexpression and in blue for underexpression relative to wild type, and represented numerically at the bottom-right corner of each panel. Non-differentially expressed protein groups and transcripts are shown in grey. For SILAC-based MS data (A-C) Venn diagram on the top right shows the overlap between the protein groups quantified in each mutant and wild type (protein level FDR are shown in parentheses).

For proteome profiling we used a variation of the stable isotope labelling by amino acids in cell culture (SILAC) method called “spike-in” SILAC [32], where heavy SILAC labelling is used only to produce a reference proteome (in our case mixed stages of wild type; S2 Fig). We quantified between 1840 and 4725 protein groups (proteins sharing same identified peptides [33]). Between 73 and 192 protein groups were found to be differentially expressed (|FC| > 1.5 and *P*-value < 0.05) between the various CPEB mutants and wild type at the corresponding developmental stage in three biological replicates (Fig 4A–4C). We did not detect any of the CPEB family members at the protein level. This is not surprising as *C*. *elegans* CPEB proteins are of low abundance (between 0.02-0.26 ppm based on PaxDb version 4 [34]) and thus unlikely to be detected via shotgun mass spectrometry (MS) after SILAC labelling (SILAC-based MS).

In parallel, we also performed transcriptome profiling of wild-type and CPEB mutant animals by RNA sequencing (RNA-seq) to complement the proteomics analysis (S2 Fig). Between 2840 and 4870 transcripts were found to be differentially expressed (|FC| > 1.5 and BH-adjusted *P*-value < 0.01) between the various CPEB mutants and wild type at the corresponding developmental stage in three biological replicates (Fig 4D–4F). The correlation between changes in proteome and transcriptome abundance in various CPEB mutants was generally weak (ranging between 0.07–0.22; S3 Fig). This is not surprising as CBEPs in other model systems are known to affect translation much more than mRNA stability. However, further factors such as indirect interactions or simply technical or biological noise can also contribute to low correlation.

We observed no increase in the mRNA abundance of the CPEB family members in the various CPEB mutants relative to wild type, with the exception of *cpb-2* mutants, where we observed a 2-3 fold increase in the transcript levels of the other three CPEB family members (Fig 3E). This suggests that there are no general compensatory mechanisms between the various CPEB family members.

### CPEB proteins in *C*. *elegans* tend to have distinct effects on gene expression

**Fig 5:**
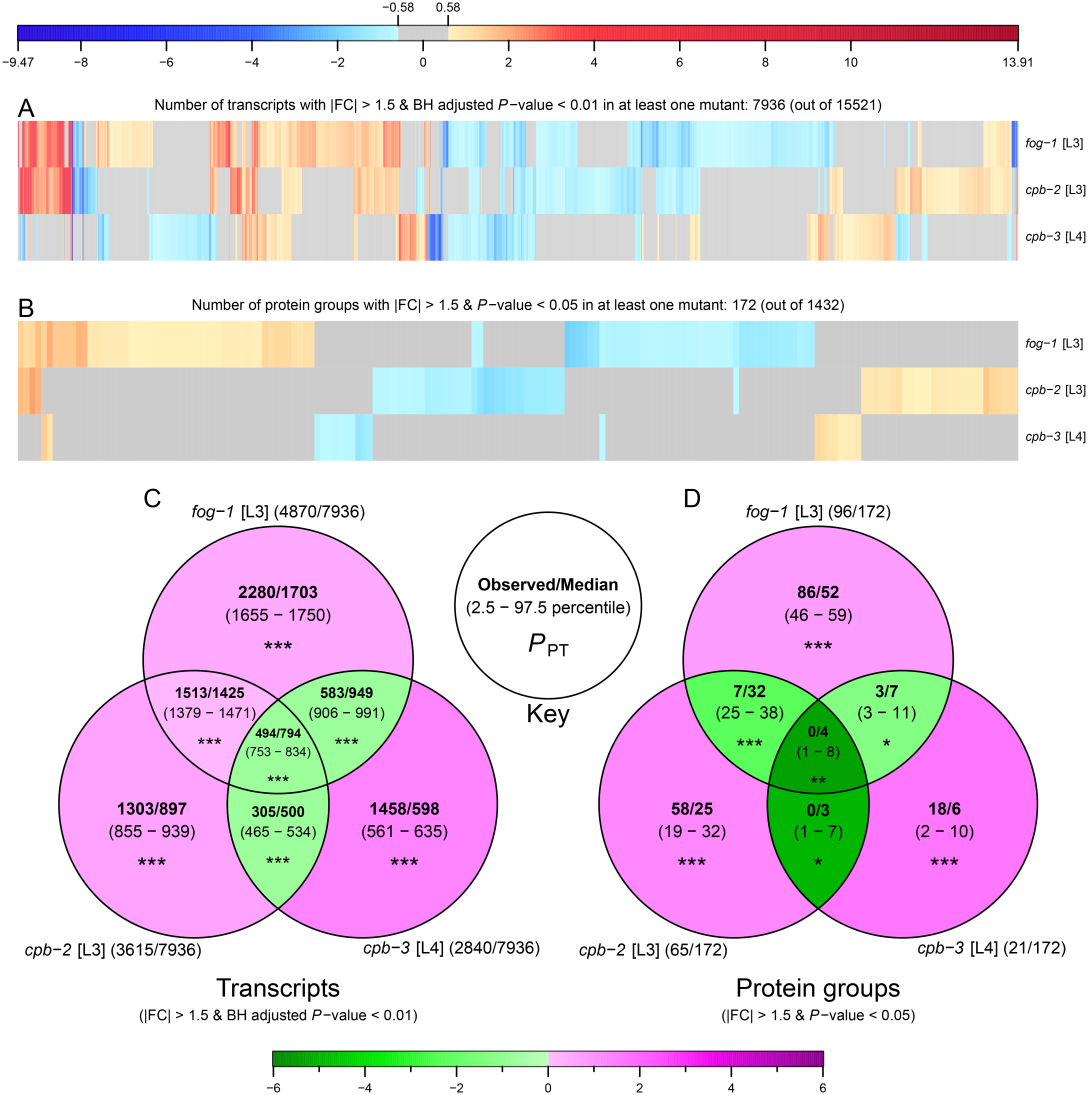
CPEB mutants tend to have distinct effects on gene expression. (A-B) Heat maps representing the differential expression of transcripts (A) and protein groups (B) in CPEB mutants relative to wild type. Columns of heat maps were clustered using functions “dist” and “hclust” from the R package “stats” (version 3.2.4) using “euclidean” distance and “complete” method. Blue and red shades represent statistically significant log_2_ scaled fold changes, grey colour is used for changes below the absolute fold change cut-off of 1.5 (~ 0.58 on log_2_ scale) or above the statistical significant cut-off. Only transcripts and protein groups quantified in all three mutants with differential expression in at least one mutant were considered. (C-D) Overlap between differentially expressed transcripts (C) and protein groups (D) in CPEB mutants. Each set shows (see key) observed value and median along with the 2.5 to 97.5 percentile range in parentheses, calculated from the random permutation for 10000 iteration. Green and magenta shades in each set represent the log_2_ scaled fold changes of observed value relative to median of permutation distribution. Asterisks denote permutation test *P*-values (*P*_PT_): *P ≤ 0.05; **P ≤ 0.01; ***P ≤ 0.001. For each set the total number of differentially expressed transcripts or protein groups out of total transcripts or protein groups used in the analysis is shown in parentheses beside set names.

As CPEB protein homologs have a high degree of sequence identity in their RNA-binding domains (RRMs and C/H domain), one could expect the *C*. *elegans* CPEB family members to bind to similar consensus sequences and thus show a certain degree of overlap in their target mRNAs. However, *C*. *elegans* CPEB proteins are also known to have non-redundant functions in germ cell differentiation pathways (with CPB-1 and FOG-1 acting in spermatogenesis; and CPB-3 being required for oogenesis) and germ line apoptosis. Because of these considerations, we analysed to what extent the changes in gene expression at the transcriptome and proteome levels would overlap in the various CPEB mutants. Overall, the three CPEB mutants had few differentially expressed genes in common (much less overlap than predicted by chance; Fig 5), both at the transcriptomics and proteomics levels. For example only 494 transcripts were differently expressed in all three mutants whereas 794 transcripts were expected by chance (Fig 5C).

Given the role of the CPEB proteins in germ cell differentiation, we next focused on genes that have previously been shown to be specifically enriched during oogenesis or spermatogenesis (oogenic and spermatogenic genes, respectively) [23]. We did not observe any major changes in the protein and transcript expression levels of oogenic and spermatogenic genes (S4 and S5 Figs and S1 Table). This observation is not surprising, as we analysed the mutants one developmental stage prior to the fully expressed phenotype. Nevertheless, consistent with the reported role of *cpb-3* in oogenesis, we observed that oogenic transcripts were as a group underexpressed in *cpb-3* mutants (S1 Table).

**Fig 6:**
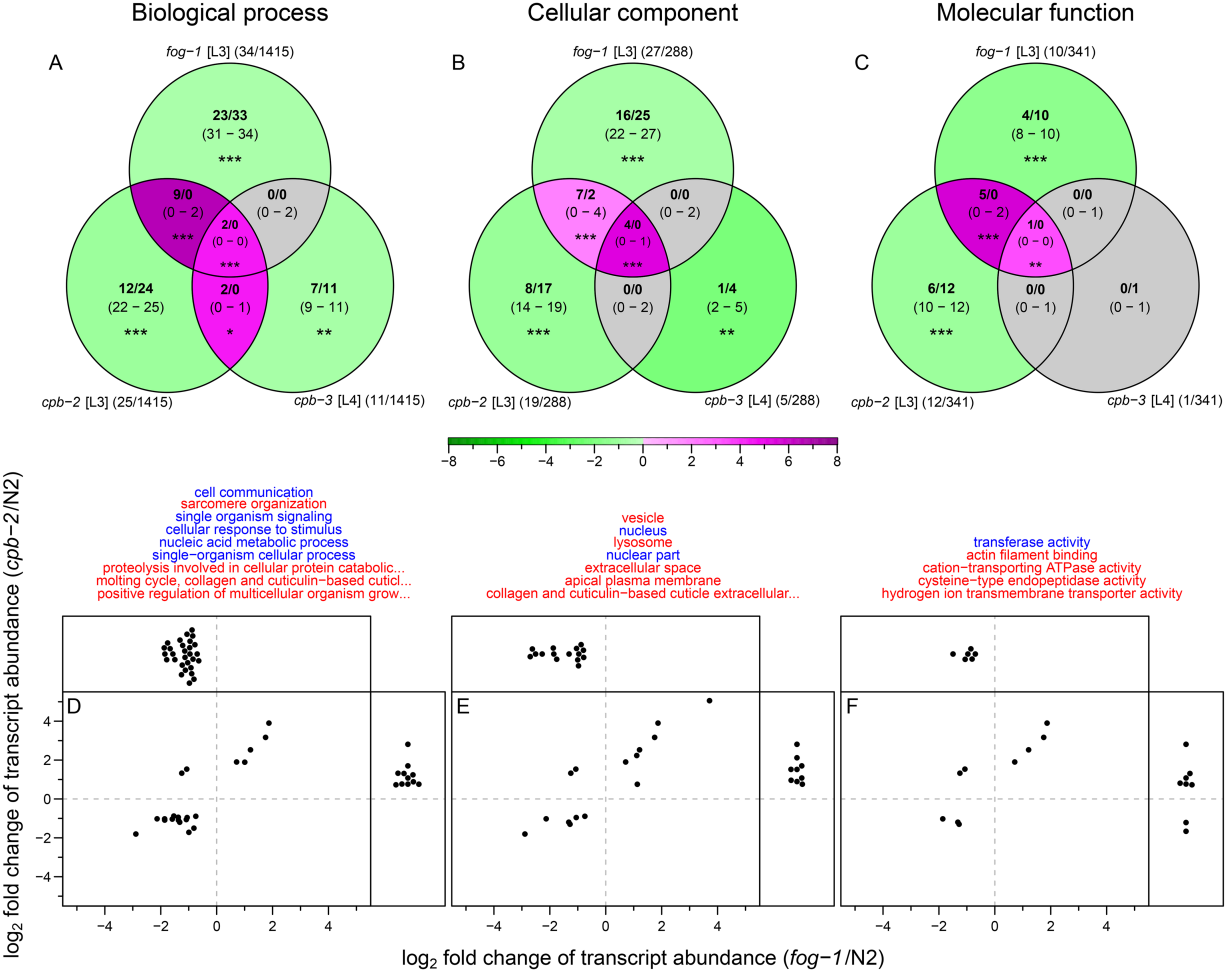
GO terms based on differentially expressed transcripts show a significant overlap between *fog-1* and *cpb-2* mutants relative to wild type. (A-C) Overlap between (A) biological process (BP), (B) cellular component (CC), and (C) molecular function (MF) ontology terms in CPEB mutants (see S6 Fig). Each set shows (see key in Fig 5) observed value and median along with the 2.5 to 97.5 percentile range in parentheses, calculated from the random permutation for 10000 iteration. Green and magenta shades in each set represent the log_2_ scaled fold changes of observed value relative to median of permutation distribution; grey colour is used for nonsignificant differences. Asterisks denote permutation test *P*-values (*P*_PT_): *P ≤ 0.05; **P ≤ 0.01; ***P ≤ 0.001. For each set the total number of significant GO terms out of total GO terms used in the analysis is shown in parentheses beside set names. (D-F) Expression profile of transcripts associated with (D) BP, (E) CC, and (F) MF ontology terms (listed on top). Each dot represents one transcript associated with the GO terms found to significantly changed (enriched or depleted) in both *fog-1* and *cpb-2* mutants (in panels A-C). Transcripts that were quantified only in one mutant background are shown on the top or the side panels, transcripts quantified in both backgrounds are shown in the main panels. GO terms enriched in both *fog-1* and *cpb-*2 mutants are coloured in red, those depleted in both *fog-1* and *cpb-*2 mutants are coloured in blue.

To further explore the differentially expressed genes (both at the transcript and protein levels) in the various CPEB mutants, we performed enrichment or depletion analysis of gene ontology (GO; [35]) terms (S6 and S7 Figs and S2 Table). A small number of GO terms based on differentially expressed transcripts were found to be enriched or depleted both in *fog-1* and *cpb-2* mutants (more than expected by chance; Fig 6). On the other hand GO terms based on differentially expressed protein groups showed no significant overlap (S7 Fig).

Taken together our observations show that loss of CPEB family members caused distinct changes in expression patterns, consistent with the hypothesis that they play different, specific roles in the *C*. *elegans* germ line.

## Conclusion

Here, we described a new missense allele of *cpb-3*. This mutation (*op234*) leads to various oogenesis defects, similar to those described previously by Hasegawa et al. for the putative null alleles *bt17* and *tm1746* [19]. Moreover, we showed that loss of *cpb-3* function give rise to higher physiological germ cell apoptosis, likely in response to oogenic defects in *cpb-3* mutants.

We also performed transcriptomics and proteomics analyses of the *fog-1*, *cpb-2*, and *cpb-3* mutants to elucidate the role of CPEB proteins in *C*. *elegans*. We found significant changes in transcript and protein abundances in all three CPEB mutants relative to wild type. Further analysis of differentially expressed genes showed that although the CPEB proteins have a high degree of sequence level similarity, they have distinct overall effects on gene expression (both at the transcript and protein levels).

Taken together, our study has helped to further define the role of the CPEB family members in the regulation of germ cell differentiation in *C*. *elegans*. Further studies, including the identification of binding sequences and target mRNAs via cross-linking and immunoprecipitation (CLIP) will be required to further understand the distinct roles of this gene family in *C*. *elegans*.

## Materials and methods

### Strains and mutations

For this study, the following mutations were used: LG I: *fog-1(q253ts)*, *unc-11(e47)*, *unc-57(ad592)*, *dpy-5(e61)*, *cpb-3(op234)*, *unc-87(e1216)*, *gla-3(op216)*, *unc-101(m1)*; LG II: *cpb-2(ok1772)*; LG III: *clk-2(mn159ts)*, *cpb-1(tm2821)*; LG IV: *ced-3(n717)*; LG V: *egl-1(n1084 n3082)*. The N2 (Bristol) strain was used as wild type. *cpb-3(op234) I* is described here and all other mutations are described in WormBase (http://www.wormbase.org) [36]. Strain EV181 *cpb-1(tm2821) III/hT2[bli-4(e937) let-*?*(q782) qIs48] (I;III)*, was received from Christian Eckmann from the Martin Luther University, Germany. Unless otherwise stated, all the strains were grown as previously described [37] on nematode growth medium (NGM) agar plates seeded with *Escherichia coli* strain OP50 at 20°C (wild-type and *fog-1* mutant worms harvested at developmental stage L3 were grown at 25°C; *fog-1* mutants were maintained at 15°C). *E*. *coli* strain AT713 (lysine/arginine auxotroph) was used for SILAC labelling.

### Isolation and cloning of *op234*

The *op234* allele was independently isolated from a forward genetic screen for mutants with increased germ cell apoptosis, as previously described [38]. The *op234* mutant was crossed back to the wild type three times before further analysis.

The *op234* allele was mapped close to the middle of chromosome I by two-factor mapping using the marker *dpy-5*. The position of op*234* was further refined by the ability of the mutation to complement the following chromosomal deficiencies: *sDf4*, *qDf16*, and *mnDf111*, and by three-factor mapping using the following marker combinations: *unc-11 dpy-5*, *unc-57 dpy-5*, *dpy-5 unc-87*, and *dpy-5 unc-101*. These analyses placed *op234* closer to *dpy-5* than *unc-87*. SNP mapping further refined the position of *op234* to a roughly 46 kb region covered by cosmids B0414 and C32F10, which includes 12 genes. RNAi was performed on all 12 genes in this interval as previously described [38]. We sequenced the PCR amplified product of the B0414.5 locus from wild-type and *op234* mutant worms to determine the molecular changes induced by *op234*.

### Apoptotic cell corpse and brood size counting

Between 15 and 20 synchronized hermaphrodites (30 h post L4/adult molt) from different genotypes (Fig 1C and 1D) were used to count germ cell corpses using differential interference contrast (DIC) microscopy as previously described [39]. Brood size analysis was performed as previously described [40].

### SILAC labelling

*C*. *elegans* strains were metabolically labelled at lysine and arginine by feeding either light-labelled lysine (^12^C_6_, ^14^N_2_ L-Lysine; Lys0) and arginine (^12^C_6_, ^14^N_4_ L-Arginine; Arg0) or heavy-labelled lysine (^13^C_6_, ^15^N_2_ L-Lysine; Lys8) and arginine (^13^C_6_, ^15^N_4_ L-Arginine; Arg10) bacteria, using the SILAC protocol as previously described [41–43]. Briefly, ~ 20000 synchronized L1 larvae were transferred to NGM (without peptone; 3 g/L NaCl, 20 g/L bacto-Agar, 5 mg/L cholesterol, 25 mM K_2_PO_4_, 1 mM MgSO_4_, 1 mM CaCl_2_) agar plates seeded with either light- or heavy-labelled bacteria.

To perform spike-in SILAC, mixed stages of wild-type worms were labelled with heavy lysine and arginine (heavy-SILAC sample) and used as reference sample. The heavy-SILAC sample was harvested after two generations (see S8 Fig for the labelling efficiency), and the protein extract was divided into single use aliquots of 150 μg and stored at -80°C till further use. Additionally the wild-type and CPEB mutant worms were labelled with light lysine and arginine (light-SILAC sample), and harvested after two generations at different developmental stages (*fog-1* and *cpb-2* at L3; *cpb-3* at L4; and N2 at L3, L4, and YA) in biological triplicates.

### Proteomics

#### Protein extraction, digestion, and peptide pre-fractionation

Protein extraction was done as previously described [44]. 150 μg of total worm protein from three biological replicates of each light-SILAC sample was mixed with one aliquot (150 μg of total worm protein) of heavy-SILAC sample and digested with trypsin as previously described [44].

Peptide samples were pre-fractionated by hydrophilic interaction liquid chromatography (HILIC) on an Agilent 1200 series HPLC system using a YMC-pack polyamine II column (250 mm × 3 mm ID, particle size 5 μm, pore size 12 nm) at a flow rate of 0.5 ml/min into 26 fractions (pooled to 11 final fractions). The previously described [44] buffer composition and elution gradient profile was used with the total run time reduced to 60 min.

#### Mass spectrometry

After peptide pre-fractionation, peptide fractions were desalted by using ZipTip C18 (Millipore) as previously described [44].

Liquid chromatography-tandem mass spectrometry (LC-MS/MS) measurements were performed on a Q Exactive mass spectrometer (Thermo Scientific) coupled with an EASY-nLC 1000 chromatography instrument (Thermo Scientific). Peptides were applied on a self-packed C-18 column (ReproSil-Pur 120 C18-AQ, 1.9 μm, 150 × 0.075 mm; Dr. Maisch GmbH) at 50°C in the loading solvent (3% ACN, 0.1% FA), and were eluted with a gradient between solvent A (water with 0.1% FA; Biosolve) and solvent B (ACN with 0.1% FA; Biosolve) at a flow rate of 300 nl/min. The gradient profile was 2%-10% solvent B between 0-1 min, 10%-30% solvent B between 1-76 min (or 2%-5% solvent B between 0-1 min, 5%-30% solvent B between 1-76 min for fraction number 13-16), 30%-50% solvent B between 76-80 min, 50%-98% solvent B between 80-84 min and 98% solvent B between 84-90 min followed by column re-equilibration to 2% solvent B in a total 90 min run.

Eluted peptides were directly ionized by electrospray ionization (ESI) and transferred into the Q Exactive orifice using the Digital PicoView (DPV-550; Newobjective) nanospray source. The mass spectrometer was operated in data dependent acquisition (DDA) mode with one full scan in Orbitrap (Scan range = 300-1700 m/z, Resolution = 70000 (at 200 m/z), AGC target = 3 × 10^6^), followed by higher-energy collisional dissociation (HCD) fragmentation of the twelve most intense ions (Charge exclusion = unassigned, 1; NCE / stepped NCE = 25; Dynamic exclusion = 30 s), and acquisition of MS/MS spectra in Orbitrap (Scan range = 200-2000 m/z, Resolution = 17500 (at 200 m/z), AGC target = 5 × 10^4^). The summary of the MS methods is available from the ProteomeXchange Consortium, dataset identifier PXD004104.

#### Proteomics data analysis

We used the MaxQuant software (version: 1.5.0.30) [33,45] for identification and quantification of protein groups from SILAC-based MS data. In each MaxQuant run, all raw data files belonging to one sample were analysed as a single “Parameter group” (Group 0), further categorized into biological replicates referred as “Experiment”, where each “Experiment” contains 11 “Fractions” (first biological replicate for *cpb-2* sample was lost during data acquisition, hence *cpb-2* sample was analysed with only two biological replicates.)

The *C*. *elegans* protein database wormpep242 (downloaded on April 2014 with 27078 entries) combined with 261 common MS contaminants (yielding a total of 27339 entries) was used for peptide spectrum matching using the Andromeda search engine [46] from MaxQuant. This forward database was concatenated with a decoy database generated by MaxQuant (Decoy mode = Revert, Special AAs = KR, and Include contaminates = checked) prior to the search, to facilitate the calculation of false discovery rate (FDR) [47]. Protein groups assigned to contaminants were also counted as forward hits for FDR calculations. Default search parameters for the Oribtrap instrument type were used with the following search settings: Fixed modifications = none; Variable modification = acetylation of the protein N-terminus, deamidation of asparagine and glutamine, and oxidation of methionine; Digestion mode = Specific; Enzyme = Trypsin with one maximum missed cleavage. SILAC labelling pairs (Heavy labels = Arg10 and Lys8; maximum of three labelled amino acids per peptide) were extracted from the isotope patterns with “Re-quantify” and “Match between run (with default settings)” enabled.

Protein quantification was performed using both unique and razor peptides (Peptides for quantification = unique + razor) with the above mentioned variable modifications, and including only proteins with at least two SILAC pairs (Min. ratio count = 2). Protein level FDR of 5% was used to export quantification results.

For each sample a further downstream analysis (based on proteinGroups.txt file) was done using R software environment [48]. First all contaminants and decoy (reversed) protein groups were removed and then for each sample only protein groups with normalised H/L ratios in at least two biological replicates were considered for calculating the fold change of protein groups between mutant and wild-type samples at the same developmental stage.

Briefly, for each protein group, deconvoluted ratios (“ratio of ratio” i.e. (H/L)_wild type_/(H/L)_mutant_) for all biological replicates between mutant and wild-type samples at the same developmental stage was calculated using values in the “Ratio H/L normalized” column. Only protein groups with deconvoluted ratios in at least two biological replicates were considered further. These deconvoluted ratios were appropriately scaled to make the median equal to 1 and then transformed to log_2_ scale. *P*-values were calculated by performing one-sample student t-test (H_0_: μ = 0 and H_1_: μ ≠ 0) on log_2_ scaled deconvoluted ratios and the average of these ratios represent average log_2_ scaled fold change of protein groups (see S9–S11 Figs for reproducibility and distribution of protein groups within replicates and samples, and S3 Table for list of protein groups quantified in each sample).

### Transcriptomics

#### RNA extraction and sequencing

From three biological replicates of each light-SILAC sample (samples from same batch were used for proteomics) RNA extraction was done using TRIzol reagent (15596-026; Life Technologies) [49] in accordance with the manufacturer’s protocol and further purified by using DNA-*free* kit (AM1906; Ambion) to obtain high quality RNA for sequencing.

RNA-seq was performed at GATC Biotech (Germany; http://www.gatc-biotech.com) using “InView^™^ Transcriptome Explore” package. In brief, a random primed cDNA library generated from each sample was sequenced on Illumina HiSeq 2500 (run type = single end, read length = 50). For some samples the cDNA library was sequenced more than once to achieve a total of at least 30 million reads.

#### Transcriptomics data analysis

RNA-seq data was analysed by a count-based approach using R and Bioconductor [50] as previously described [51]. In summary, raw sequencing data files (FASTQ format) were assessed for their quality by using R package “ShortRead” (version 1.22.0) [52] and FastQC (version 0.11.2, Babraham Bioinformatics, http://www.bioinformatics.babraham.ac.uk). After quality control checks (all FASTQ files passed), RNA-seq reads were aligned to the *C*. *elegans* reference genome WBcel235 (release-76; downloaded on September 2014 from Ensembl) using aligner TopHat (version 2.0.9) [53]. For all samples mapped reads were counted by the *htseq-count* script from HTSeq (version 0.6.1p1) [54] to generate a count table. Counts from different sequencing runs of the same cDNA library were summed prior to the differential analysis. Finally, differential analysis of counts between mutant and wild-type samples at the same developmental stage was done using R package “edgeR” (version 3.6.8) [55] (see S12 and S13 Figs for reproducibility and distribution of transcripts within replicates and samples, and S3 Table for list of transcripts quantified in each sample).

### GO analysis

R package “org.Ce.eg.db” (version 3.2.3) [56] was used to retrieve *GO-to-genes* annotations, and genes annotated with evidence codes ND, IEA, and NR were removed prior to the analysis. GO analysis was performed individually for biological process (BP), cellular component (CC), and molecular function (MF) ontologies by using R package “topGO” (version 2.22.0) [57] with nodeSize = 10. The set of all quantified protein groups or transcripts was used as the gene universe and differentially expressed protein groups (|FC| > 1.5 and *P*-value < 0.05) or transcripts (|FC| > 1.5, BH-adjusted *P*-value < 0.01, and log_2_(CPM) > 8) were considered as interesting genes. Custom test statistic function for the two-tailed Fisher's exact test in combination with “elim” algorithm [58] was used to perform gene counts based enrichment or depletion tests (see S2 Table, for list of significantly enriched or depleted GO terms in proteomics and transcriptomics datasets).

## Supporting information

S1 Fig *C*. *elegans* contains four distantly related CPEB homologs. Unrooted phylogenetic tree showing evolutionary relationship between CPEB orthologs and paralogs across different organisms. Multiple protein sequence alignment was performed using CLUSTAL W [59] via R package “msa” (version 1.2.1) [60] with default arguments. The tree was calculated (after filtering alignment positions for at least 10% non-gap) using “dist.alignment” function from R package “seqinr” (version 3.1-3) [61] and drawn using neighbor-joining method with “nj” and “plot.phylo” functions from R package “ape” (version 3.4) [62]. Numbers on the internal node represent the bootstrapping score for 1000 iterations calculated using “boot.phylo" function from R package “ape”. *C*. *elegans* proteins are in blue. The four clades containing the vertebrate CPEB proteins are highlighted. Length of proteins is shown in parentheses beside protein names (aa: amino acids). CPEB proteins from the following organisms are shown: *C*. *elegans* (Ce), *Drosophila melanogaster* (Dm), *Spisula solidissima* (Ss), *Danio rerio* (Dr), *Xenopus laevis* (Xl), *Xenopus tropicalis* (Xt), *Mus musculus* (Mm) and *Homo sapiens* (Hs).
(TIF)

S2 Fig Overview of SILAC-based MS and RNA-seq sample preparation and data analysis pipelines. Wild-type and CPEB mutant worms were labelled with light lysine and arginine and harvested after two generations at different developmental stages (*fog-1* and *cpb-2* at L3; *cpb-3* at L4 and N2 at L3, L4 and YA; light-SILAC sample; green) in biological triplicates. These worms were used for proteome and transcriptome analyses. Mixed stages of wild-type worms were labelled with heavy lysine and arginine (heavy-SILAC sample; red). For proteomics, protein extracts from light- and heavy-SILAC samples were mixed in 1:1 ratio (yellow), before trypsinization, HILIC, and MS. For transcriptomics, total RNA was extracted from each light-SILAC sample and submitted for RNA-seq. See Materials and methods for details.
(TIF)

S3 Fig Weak correlation between changes in proteome and transcriptome abundance in CPEB mutants relative to wild type. Scatter plot between log_2_ scaled fold change in protein group and transcript abundances in *fog-1* (A), *cpb-2* (B) and *cpb-3* (C) mutants relative to wild type. Data points are coloured as follows: blue for significant at protein level (*P*-value < 0.05), green for significant at transcript level (BH-adjusted *P*-value < 0.01), orange for significant at protein and transcript levels, and grey for others. Number of data points is denoted by N and Pearson correlation coefficient is denoted by r (values in black correspond to all data points).
(TIF)

S4 Fig Overall protein group expression profile. Distribution of protein group expression levels in *fog-1* (A and D), *cpb-2* (B and E), and *cpb-3* (C and F) mutants relative to wild type. (A-C) Kernel density plots for oogenic (magenta), spermatogenic (blue), and all (black) protein groups. (D-F) Empirical cumulative density plots for oogenic (magenta), spermatogenic (blue), and other remaining (grey) protein groups. *P*-value from two-tailed Kolmogorov-Smirnov test between oogenic or spermatogenic protein groups and other protein groups is denoted by *P*_KS_. Vertical dashed lines represent the fold change cut-off of 1.5 (~ 0.58 on log_2_ scale). Number of data points in each category denoted by N.
(TIF)

S5 Fig Overall transcript expression profile. Distribution of transcript expression levels in *fog-1* (A and D), *cpb-2* (B and E), and *cpb-3* (C and F) mutants relative to wild type. (A-C) Kernel density plots for oogenic (magenta), spermatogenic (blue), and all (black) transcripts. (D-F) Empirical cumulative density plots for oogenic (magenta), spermatogenic (blue), and other remaining (grey) transcripts. *P*-value from two-tailed Kolmogorov-Smirnov test between oogenic or spermatogenic transcripts and other transcripts is denoted by *P*_KS_. Vertical dashed lines represent the fold change cut-off of 1.5 (~ 0.58 on log_2_ scale). Number of data points in each category denoted by N.
(TIF)

S6 Fig Word clouds of the significantly enriched or depleted GO terms based on differentially expressed transcripts in CPEB mutants relative to wild type. GO analysis was performed on highly abundant, differentially expressed transcripts (|FC| > 1.5, BH-adjusted *P*-value < 0.01, and log_2_(CPM) > 8) using R package “topGO” (version 2.22.0) [57]. GO terms from BP, CC, and MF ontologies with two-tailed Fisher's exact test *P*-value < 0.05 in three CPEB mutants are shown here as word clouds using R package “GOsummaries” (version 2.4.7) [63]. In the word clouds the size of the words is proportional to -log_10_ of *P*-value within one word cloud. Terms are coloured as follows: red for enriched and blue for depleted.
(TIF)

S7 Fig GO analysis of differentially expressed protein groups in CPEB mutants relative to wild type. (A) Word clouds of the significantly enriched or depleted GO terms. GO analysis was performed on differentially expressed protein groups (|FC| > 1.5 and *P*-value < 0.05) using R package “topGO” (version 2.22.0) [57]. GO terms from BP, CC, and MF ontologies with two-tailed Fisher's exact test *P*-value < 0.05 in three CPEB mutants are shown here as word clouds using R package “GOsummaries” (version 2.4.7) [63]. In the word clouds the size of the words is proportional to -log_10_ of *P*-value within one word cloud. Terms are coloured as follows: red for enriched and blue for depleted. (B-D) Overlap between BP (B), CC (C), and MF (D) ontology terms in CPEB mutants. Each set shows (see key in Fig 5) observed value and median along with the 2.5 to 97.5 percentile range in parentheses, calculated from the random permutation for 10000 iteration. None of the overlaps was significant. For each set the total number of significant GO terms out of total GO terms used in the analysis is shown in parentheses beside set names.
(TIF)

S8 Fig Incorporation efficiency of heavy amino acids labelled by SILAC. Tukey-style box plot for heavy-labelled lysine (Lys8) and arginine (Arg10) incorporation in heavy-SILAC sample harvested after one (F_1_) and two (F_2_) generations. Heavy-SILAC samples were processed and analysed (with “Re-quantify” disabled) as described in Materials and Methods. The incorporation efficiency on the peptide level (based on peptides.txt file) was calculated using the following equation: (Intensity H/(Intensity L + Intensity H) × 100. Number of peptides (with missed cleavage = 0) used for analysis is denoted by N on the top and average incorporation efficiency is represented at the bottom of labels.
(TIF)

S9 Fig Protein abundances have high reproducibility between biological replicates (based on intensities from light isoforms). Scatter plot of protein abundances (log_10_ scaled intensities from light isoforms of protein groups) between different biological replicates of each sample. Number of data points is denoted by N and Pearson correlation coefficient is denoted by r.
(TIF)

S10 Fig Protein abundances have high reproducibility between biological replicates (based on normalised L/H ratios). Scatter plot of protein abundances (log_2_ scaled normalised L/H ratios of protein groups) between different biological replicates of each sample. Number of data points is denoted by N and Pearson correlation coefficient is denoted by r.
(TIF)

S11 Fig Distribution of protein abundances is similar in all samples. Matrix plot of protein abundances (log_10_ scaled median intensities from light isoforms of protein groups) across samples. Diagonal shows histogram of protein abundances in each sample. Scatter plots between samples are shown below the diagonal and Pearson correlation coefficients between samples are shown above the diagonal. Number of data points is denoted by N.
(TIF)

S12 Fig Transcript abundances have high reproducibility between biological replicates. Scatter plot of transcript abundances (log_2_ scaled normalised CPM + 1) between different biological replicates of each sample. Number of data points is denoted by N and Pearson correlation coefficient is denoted by r.
(TIF)

S13 Fig Distribution of transcript abundances is similar in all samples. Matrix plot of transcript abundances (log_2_ scaled normalised CPM + 1) across samples. Diagonal shows histogram of transcript abundances in each sample. Scatter plots between samples are shown below the diagonal and Pearson correlation coefficients between samples are shown above the diagonal. Number of data points is denoted by N.
(TIF)

S1 Table Distribution of oogenic and spermatogenic genes in proteome (A) and transcriptome (B) of CPEB mutants relative to wild type. Observed/expected number of gender neutral, oogenic, and spermatogenic genes found in the over- and underexpressed proteome (A) and transcriptome (B) of CPEB mutants relative to wild type. Parentheses shows the log_2_ scaled fold change of observed number relative to expected number (positive value means enrichment and negative value means depletion). Two-tailed Fisher’s exact test *P*-values are shown at the bottom of each category.
(PDF)

S2 Table Significantly enriched or depleted GO terms in proteome and transcriptome of CPEB mutants relative to wild type. (XLSX)

S3 Table Protein groups and transcripts quantified in CPEB homolog mutants relative to wild type. (XLSX)

S4 Table List of abbreviations. (XLSX)
