# Supplementary figures and images for: Differential regulation of germ line apoptosis and germ cell differentiation by CPEB family members in C. elegans
Source: PLoS One. 2017 Jul 31;12(7):e0182270. doi: 10.1371/journal.pone.0182270 (PMC5536308; doi:10.1371/journal.pone.0182270)

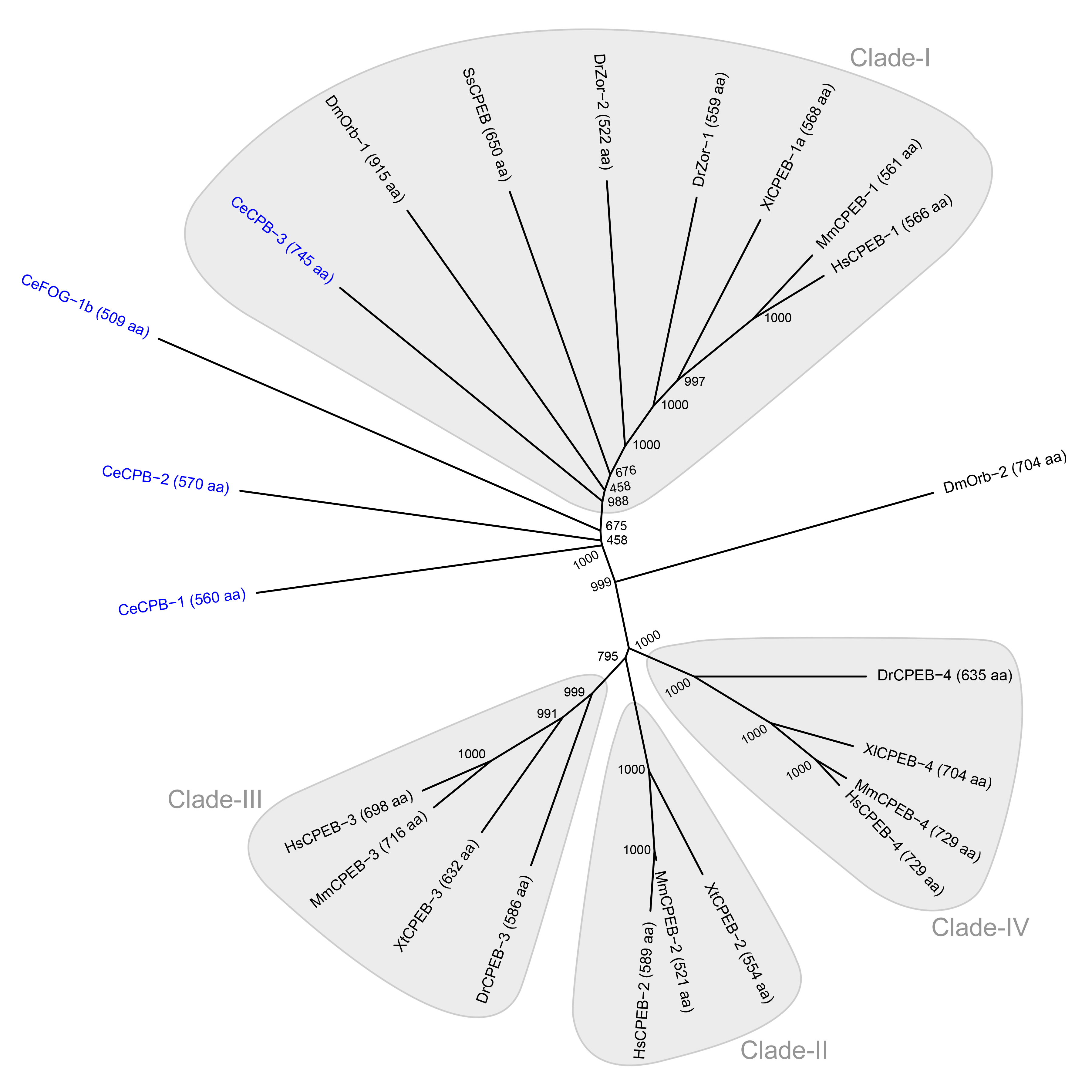

Supplement: S1 Fig — Unrooted phylogenetic tree showing evolutionary relationship between CPEB orthologs and paralogs across different organisms. Multiple protein sequence alignment was performed using CLUSTAL W [59] via R package “msa” (version 1.2.1) [60] with default arguments. The tree was calculated (after filtering alignment positions for at least 10% non-gap) using “dist.alignment” function from R package “seqinr” (version 3.1-3) [61] and drawn using neighbor-joining method with “nj” and “plot.phylo” functions from R package “ape” (version 3.4) [62]. Numbers on the internal node represent the bootstrapping score for 1000 iterations calculated using “boot.phylo" function from R package “ape”. C. elegans proteins are in blue. The four clades containing the vertebrate CPEB proteins are highlighted. Length of proteins is shown in parentheses beside protein names (aa: amino acids). CPEB proteins from the following organisms are shown: C. elegans (Ce), Drosophila melanogaster (Dm), Spisula solidissima (Ss), Danio rerio (Dr), Xenopus laevis (Xl), Xenopus tropicalis (Xt), Mus musculus (Mm) and Homo sapiens (Hs). (TIF) [file pone.0182270.s001.tif]

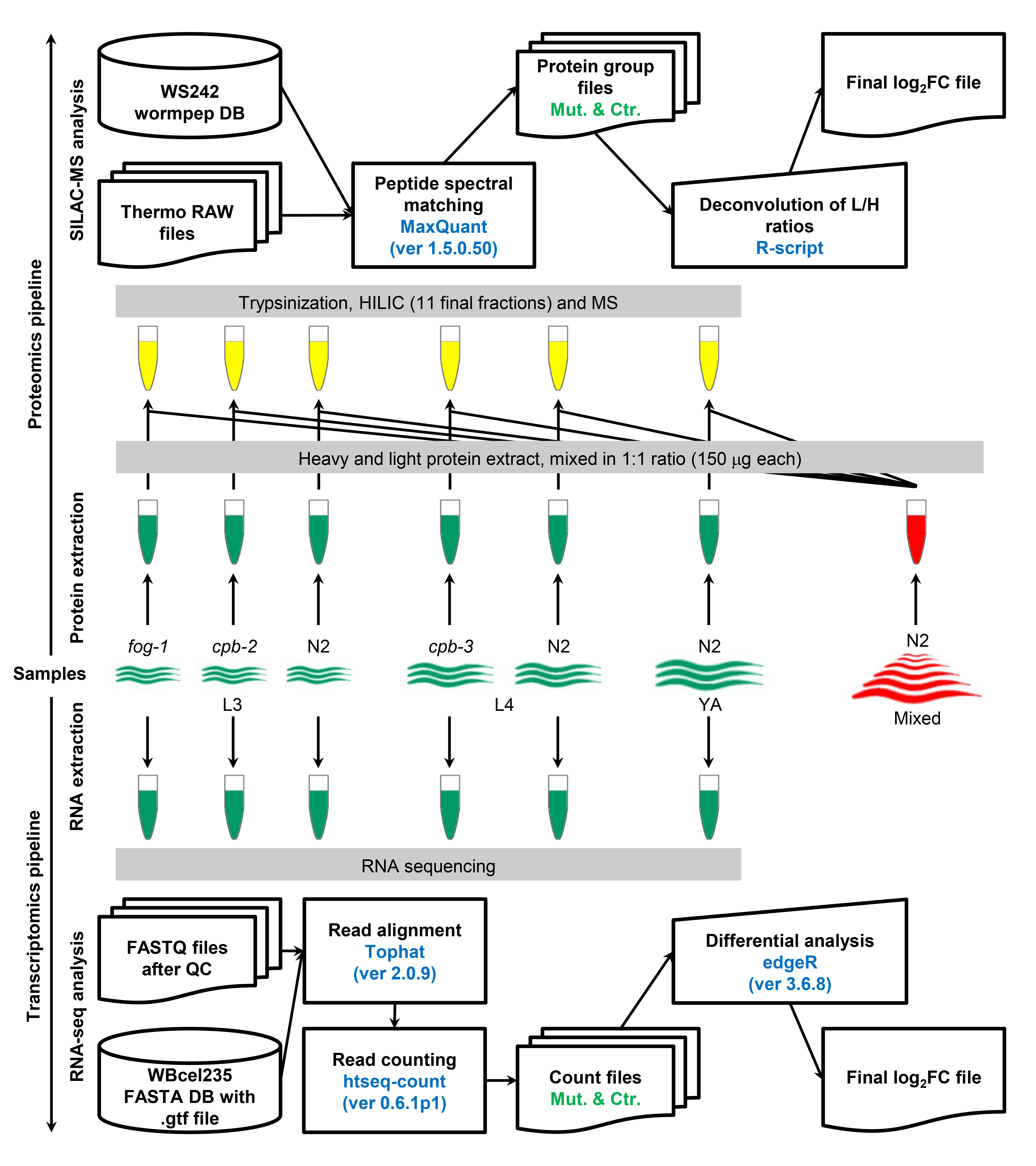

Supplement: S2 Fig — Wild-type and CPEB mutant worms were labelled with light lysine and arginine and harvested after two generations at different developmental stages (fog-1 and cpb-2 at L3; cpb-3 at L4 and N2 at L3, L4 and YA; light-SILAC sample; green) in biological triplicates. These worms were used for proteome and transcriptome analyses. Mixed stages of wild-type worms were labelled with heavy lysine and arginine (heavy-SILAC sample; red). For proteomics, protein extracts from light- and heavy-SILAC samples were mixed in 1:1 ratio (yellow), before trypsinization, HILIC, and MS. For transcriptomics, total RNA was extracted from each light-SILAC sample and submitted for RNA-seq. See Materials and methods for details. (TIF) [file pone.0182270.s002.tif]

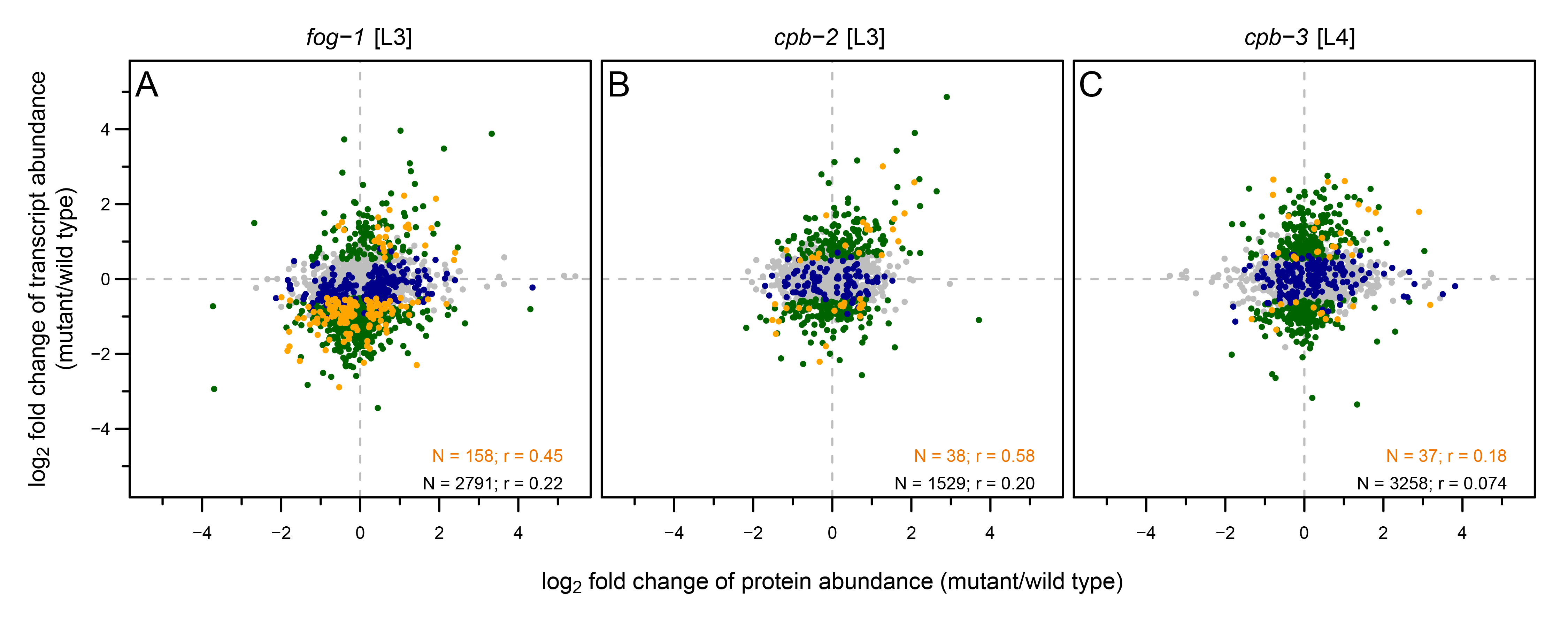

Supplement: S3 Fig — Scatter plot between log2 scaled fold change in protein group and transcript abundances in fog-1 (A), cpb-2 (B) and cpb-3 (C) mutants relative to wild type. Data points are coloured as follows: blue for significant at protein level (P-value < 0.05), green for significant at transcript level (BH-adjusted P-value < 0.01), orange for significant at protein and transcript levels, and grey for others. Number of data points is denoted by N and Pearson correlation coefficient is denoted by r (values in black correspond to all data points). (TIF) [file pone.0182270.s003.tif]

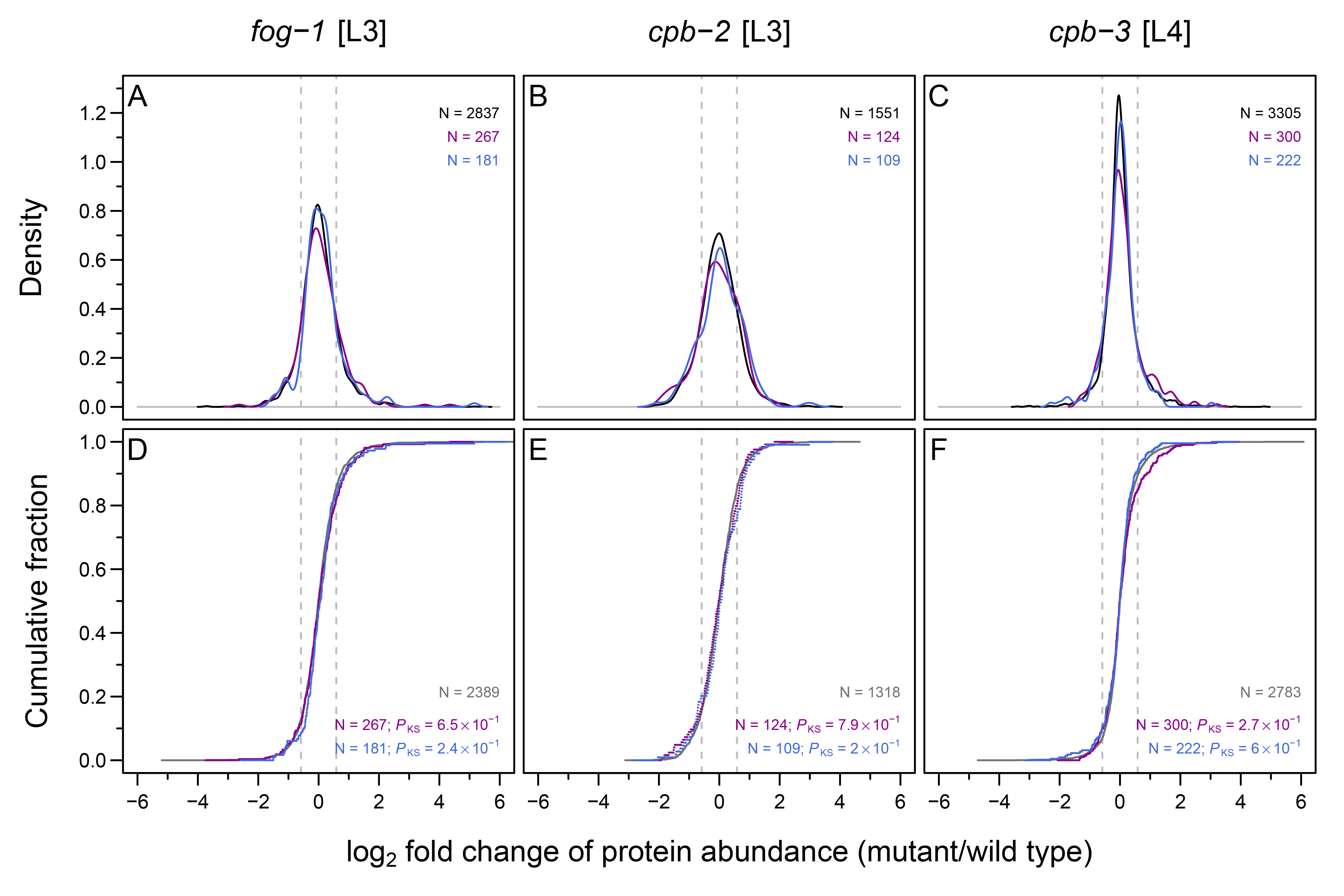

Supplement: S4 Fig — Distribution of protein group expression levels in fog-1 (A and D), cpb-2 (B and E), and cpb-3 (C and F) mutants relative to wild type. (A-C) Kernel density plots for oogenic (magenta), spermatogenic (blue), and all (black) protein groups. (D-F) Empirical cumulative density plots for oogenic (magenta), spermatogenic (blue), and other remaining (grey) protein groups. P-value from two-tailed Kolmogorov-Smirnov test between oogenic or spermatogenic protein groups and other protein groups is denoted by PKS. Vertical dashed lines represent the fold change cut-off of 1.5 (~ 0.58 on log2 scale). Number of data points in each category denoted by N. (TIF) [file pone.0182270.s004.tif]

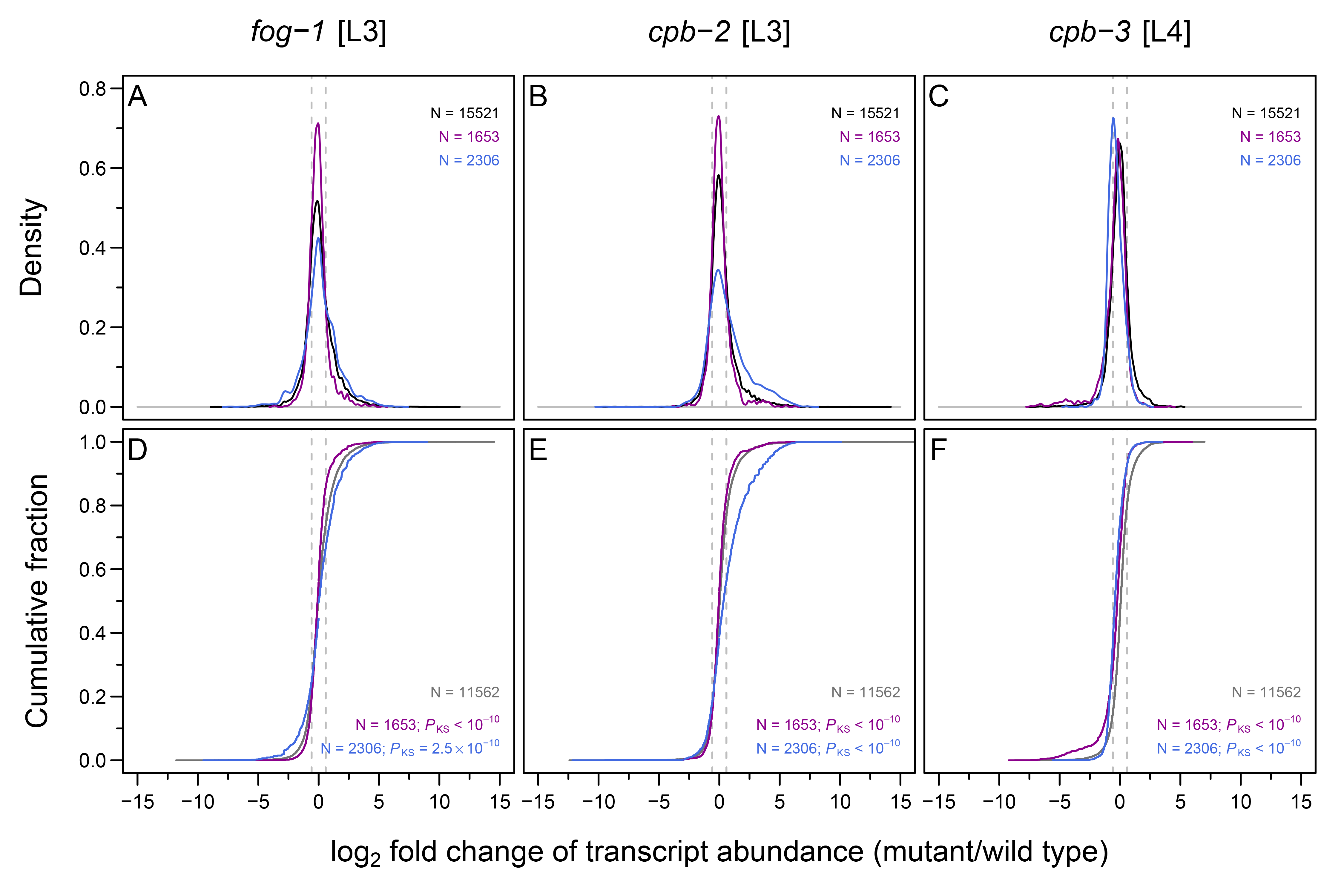

Supplement: S5 Fig — Distribution of transcript expression levels in fog-1 (A and D), cpb-2 (B and E), and cpb-3 (C and F) mutants relative to wild type. (A-C) Kernel density plots for oogenic (magenta), spermatogenic (blue), and all (black) transcripts. (D-F) Empirical cumulative density plots for oogenic (magenta), spermatogenic (blue), and other remaining (grey) transcripts. P-value from two-tailed Kolmogorov-Smirnov test between oogenic or spermatogenic transcripts and other transcripts is denoted by PKS. Vertical dashed lines represent the fold change cut-off of 1.5 (~ 0.58 on log2 scale). Number of data points in each category denoted by N. (TIF) [file pone.0182270.s005.tif]

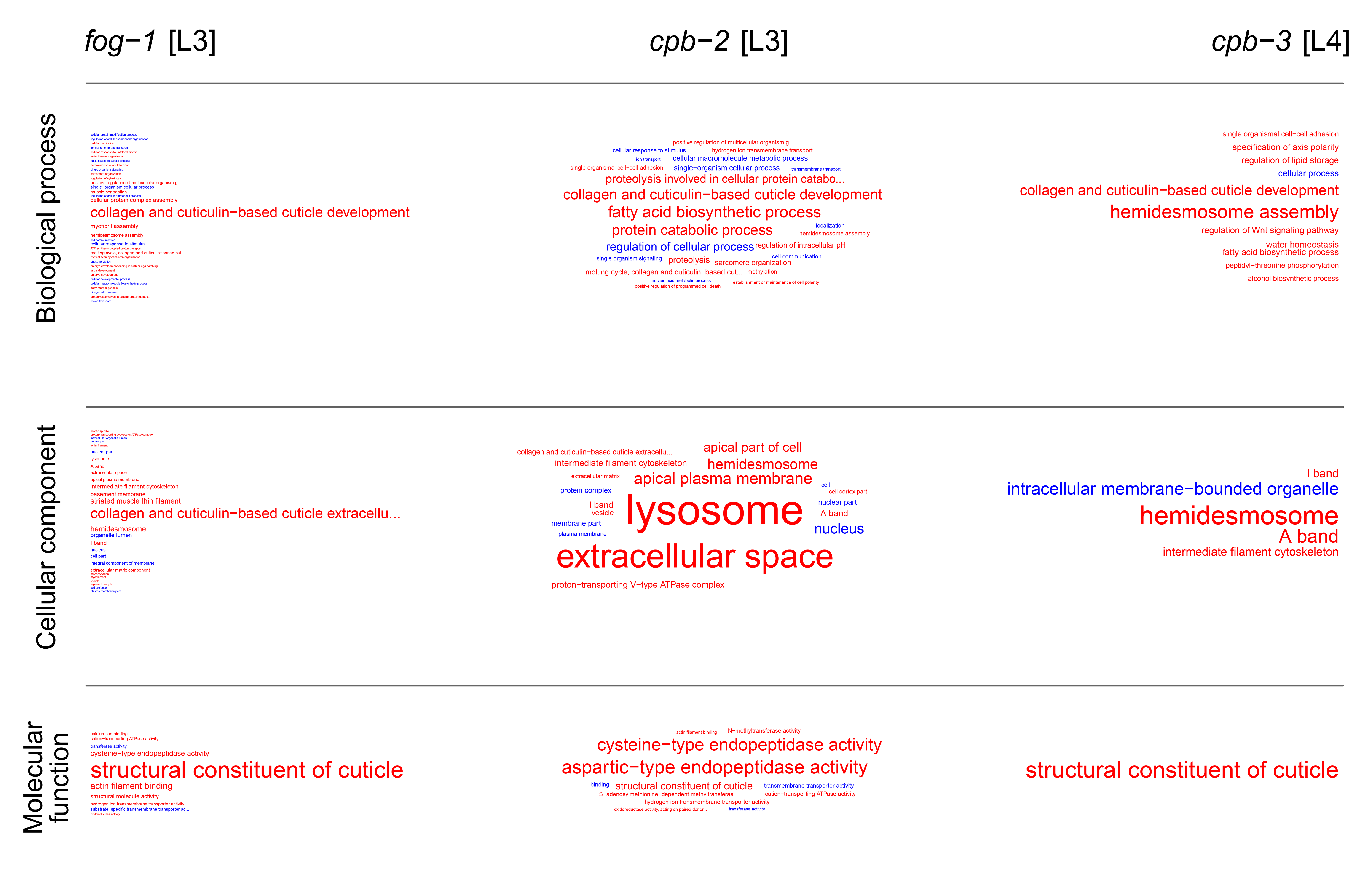

Supplement: S6 Fig — GO analysis was performed on highly abundant, differentially expressed transcripts (|FC| > 1.5, BH-adjusted P-value < 0.01, and log2(CPM) > 8) using R package “topGO” (version 2.22.0) [57]. GO terms from BP, CC, and MF ontologies with two-tailed Fisher's exact test P-value < 0.05 in three CPEB mutants are shown here as word clouds using R package “GOsummaries” (version 2.4.7) [63]. In the word clouds the size of the words is proportional to -log10 of P-value within one word cloud. Terms are coloured as follows: red for enriched and blue for depleted. (TIF) [file pone.0182270.s006.tif]

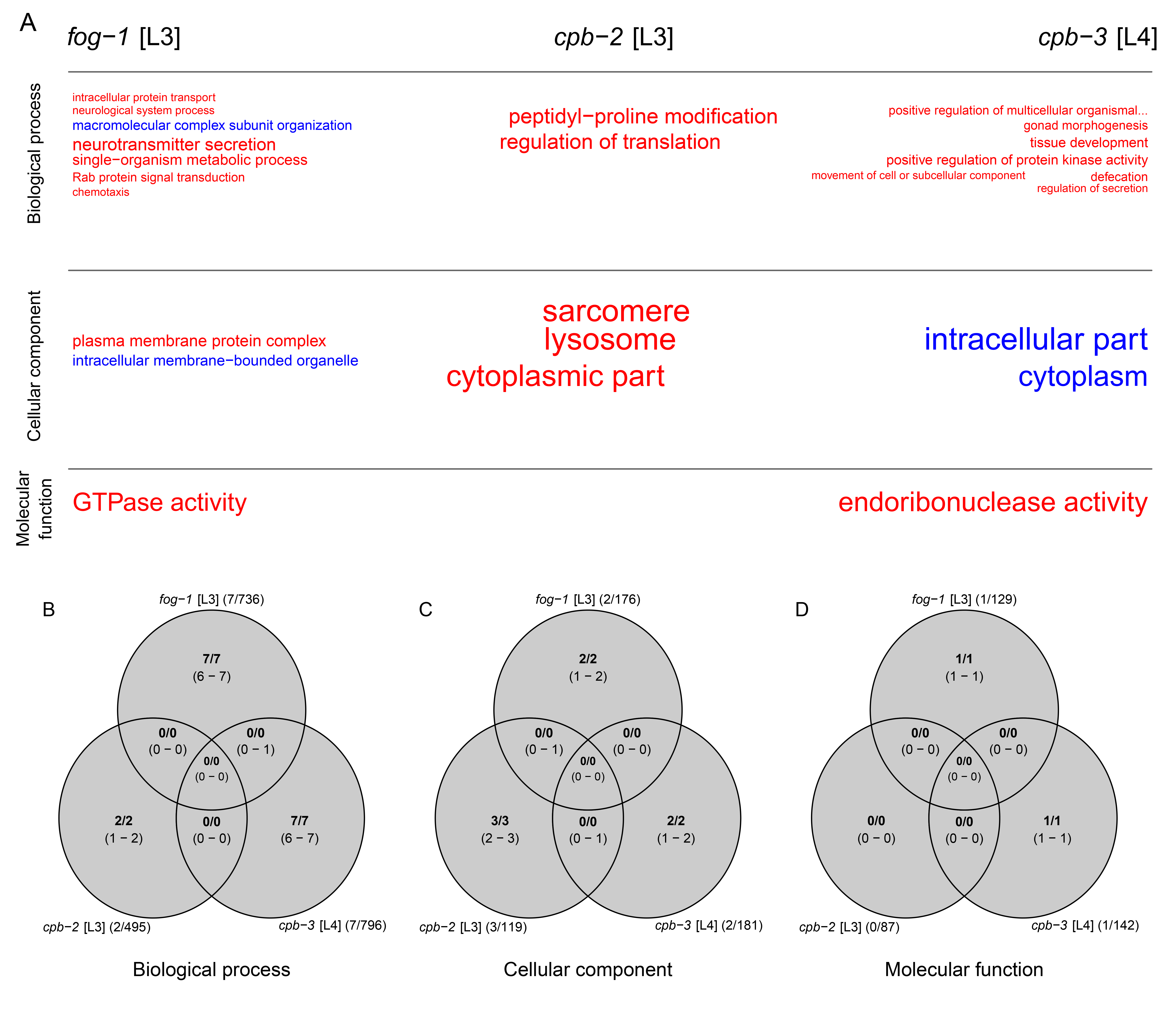

Supplement: S7 Fig — (A) Word clouds of the significantly enriched or depleted GO terms. GO analysis was performed on differentially expressed protein groups (|FC| > 1.5 and P-value < 0.05) using R package “topGO” (version 2.22.0) [57]. GO terms from BP, CC, and MF ontologies with two-tailed Fisher's exact test P-value < 0.05 in three CPEB mutants are shown here as word clouds using R package “GOsummaries” (version 2.4.7) [63]. In the word clouds the size of the words is proportional to -log10 of P-value within one word cloud. Terms are coloured as follows: red for enriched and blue for depleted. (B-D) Overlap between BP (B), CC (C), and MF (D) ontology terms in CPEB mutants. Each set shows (see key in Fig 5) observed value and median along with the 2.5 to 97.5 percentile range in parentheses, calculated from the random permutation for 10000 iteration. None of the overlaps was significant. For each set the total number of significant GO terms out of total GO terms used in the analysis is shown in parentheses beside set names. (TIF) [file pone.0182270.s007.tif]

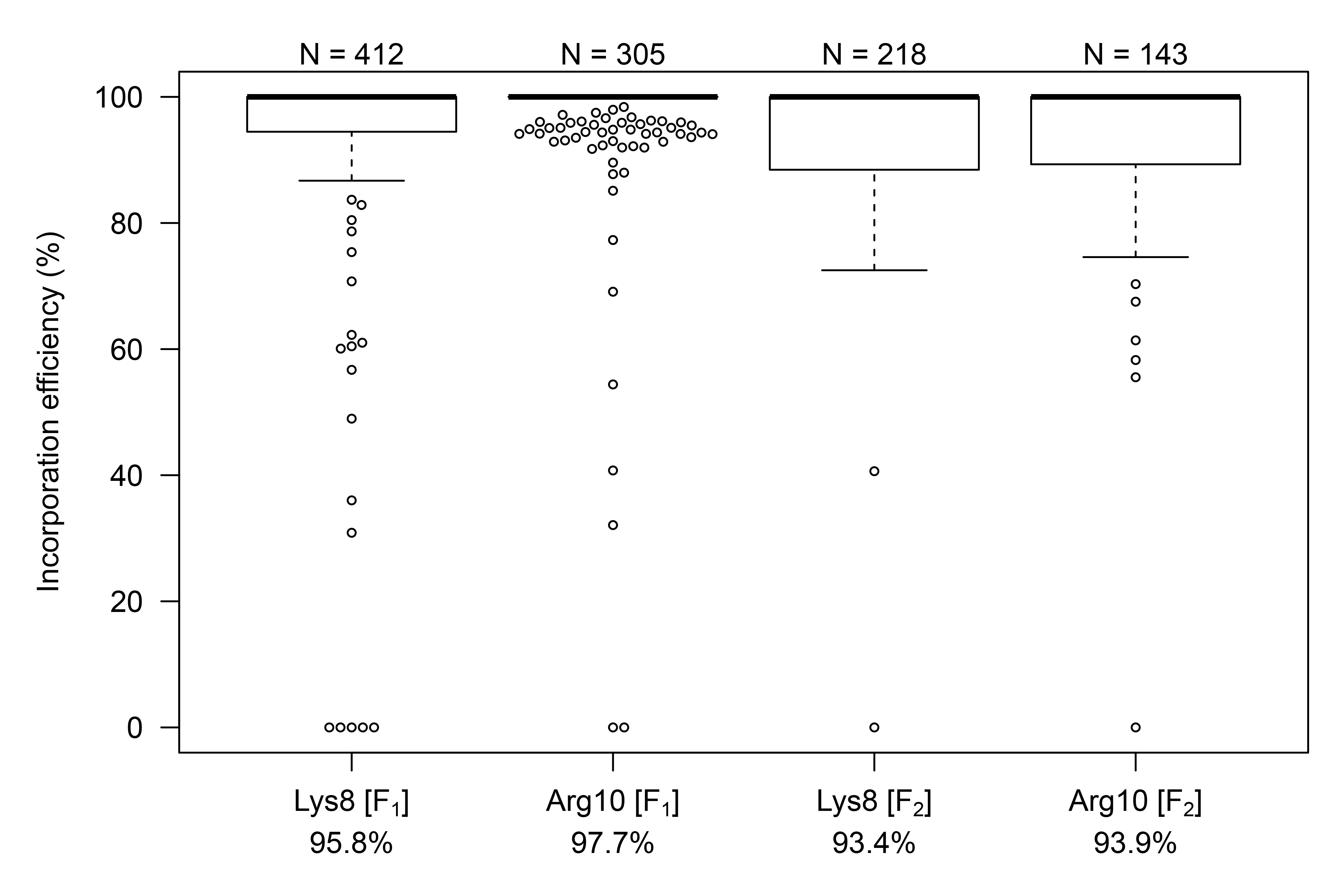

Supplement: S8 Fig — Tukey-style box plot for heavy-labelled lysine (Lys8) and arginine (Arg10) incorporation in heavy-SILAC sample harvested after one (F1) and two (F2) generations. Heavy-SILAC samples were processed and analysed (with “Re-quantify” disabled) as described in Materials and Methods. The incorporation efficiency on the peptide level (based on peptides.txt file) was calculated using the following equation: (Intensity H/(Intensity L + Intensity H) × 100. Number of peptides (with missed cleavage = 0) used for analysis is denoted by N on the top and average incorporation efficiency is represented at the bottom of labels. (TIF) [file pone.0182270.s008.tif]

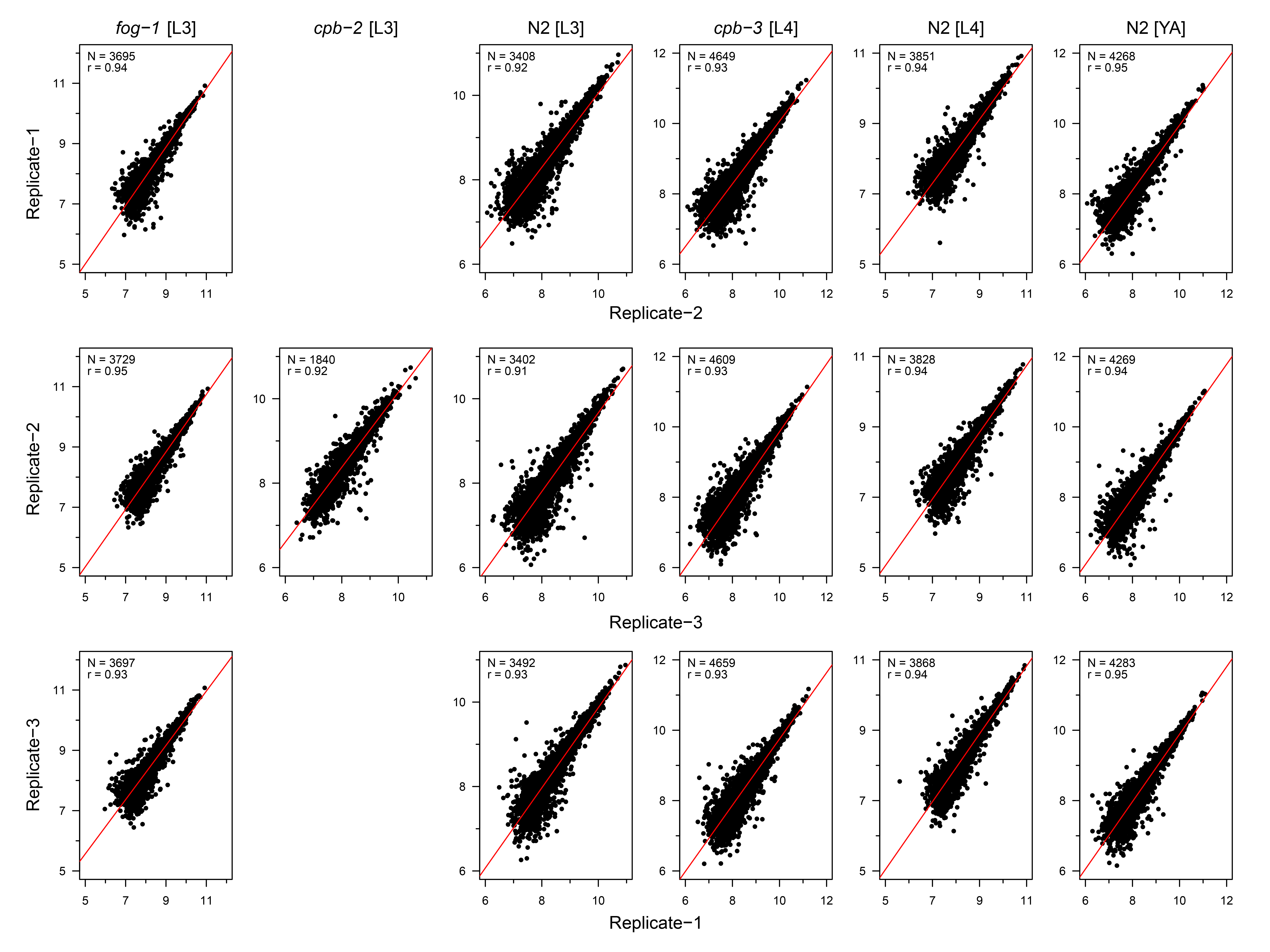

Supplement: S9 Fig — Scatter plot of protein abundances (log10 scaled intensities from light isoforms of protein groups) between different biological replicates of each sample. Number of data points is denoted by N and Pearson correlation coefficient is denoted by r. (TIF) [file pone.0182270.s009.tif]

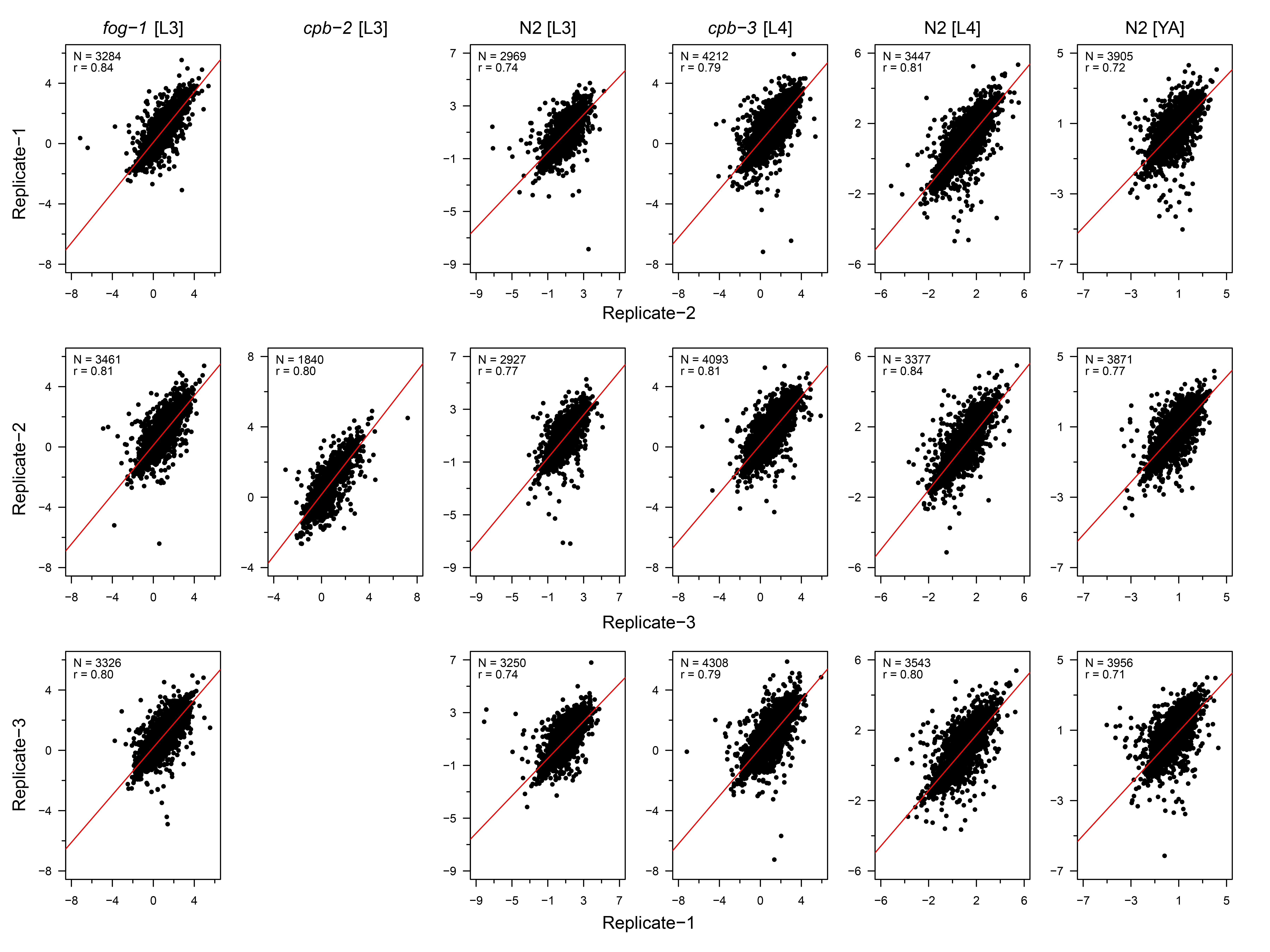

Supplement: S10 Fig — Scatter plot of protein abundances (log2 scaled normalised L/H ratios of protein groups) between different biological replicates of each sample. Number of data points is denoted by N and Pearson correlation coefficient is denoted by r. (TIF) [file pone.0182270.s010.tif]

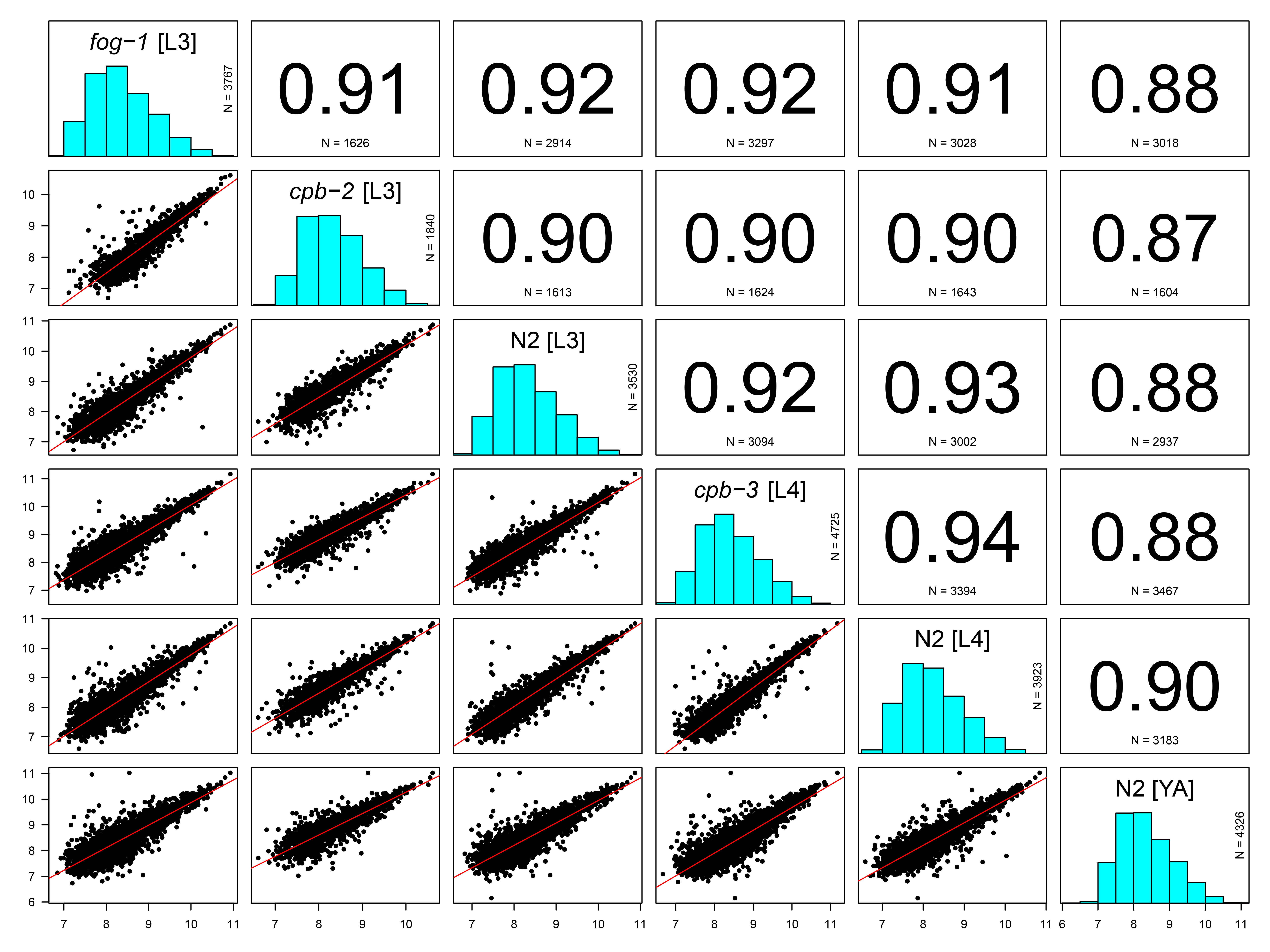

Supplement: S11 Fig — Matrix plot of protein abundances (log10 scaled median intensities from light isoforms of protein groups) across samples. Diagonal shows histogram of protein abundances in each sample. Scatter plots between samples are shown below the diagonal and Pearson correlation coefficients between samples are shown above the diagonal. Number of data points is denoted by N. (TIF) [file pone.0182270.s011.tif]

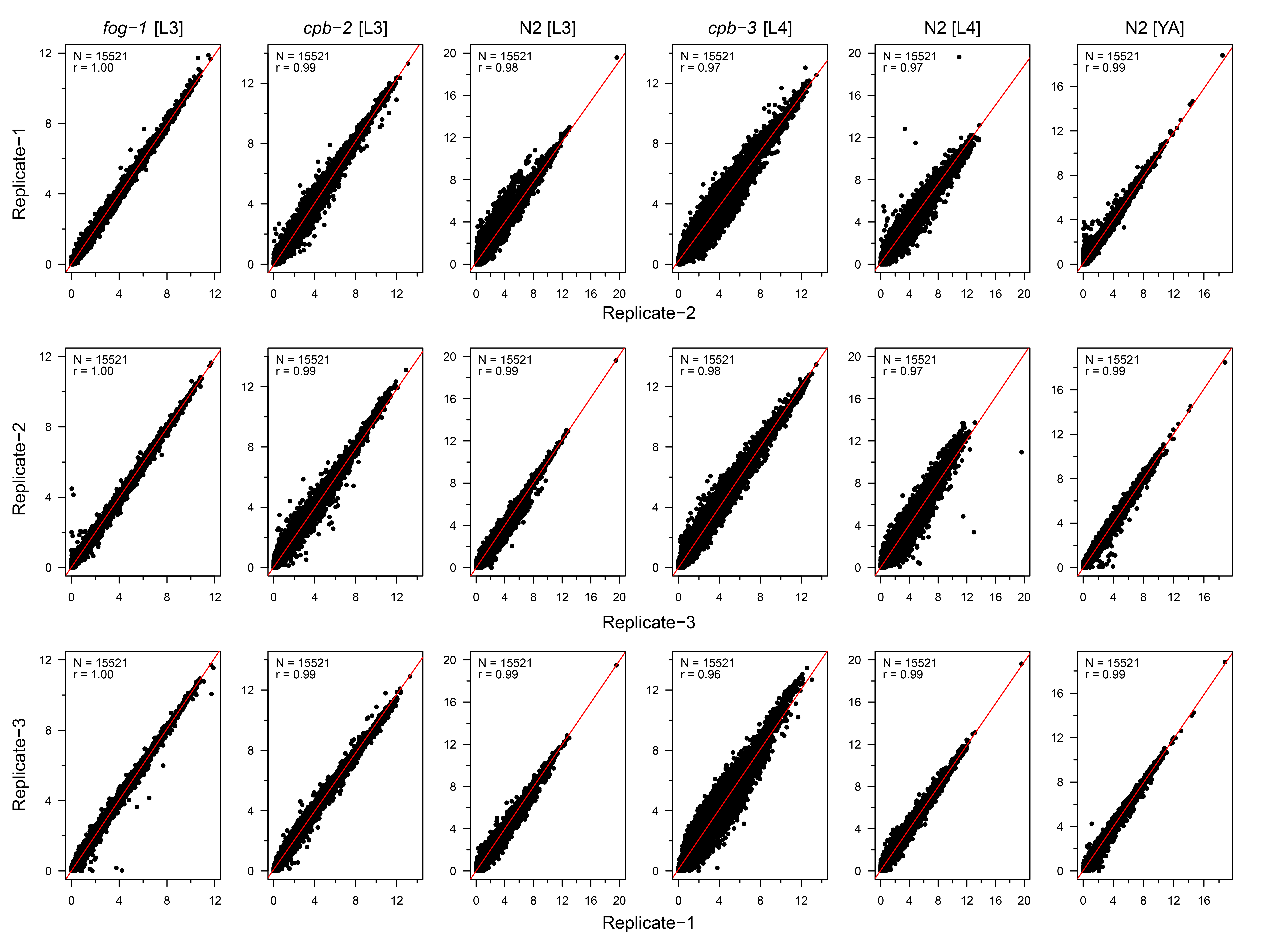

Supplement: S12 Fig — Scatter plot of transcript abundances (log2 scaled normalised CPM + 1) between different biological replicates of each sample. Number of data points is denoted by N and Pearson correlation coefficient is denoted by r. (TIF) [file pone.0182270.s012.tif]

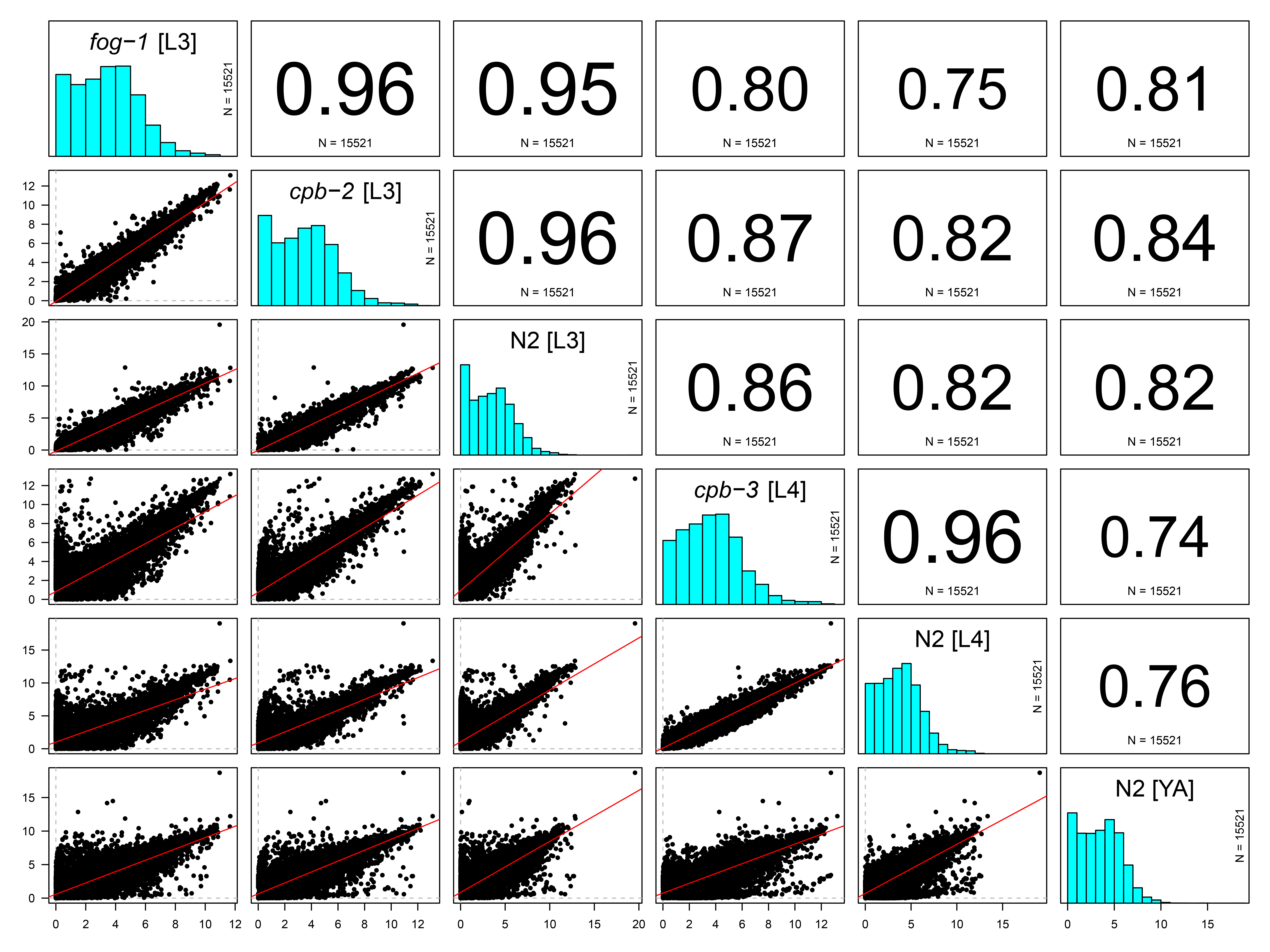

Supplement: S13 Fig — Matrix plot of transcript abundances (log2 scaled normalised CPM + 1) across samples. Diagonal shows histogram of transcript abundances in each sample. Scatter plots between samples are shown below the diagonal and Pearson correlation coefficients between samples are shown above the diagonal. Number of data points is denoted by N. (TIF) [file pone.0182270.s013.tif]
