# Supplementary material for: Differential regulation of germ line apoptosis and germ cell differentiation by CPEB family members in C. elegans
Source: PLoS One. 2017 Jul 31;12(7):e0182270. doi: 10.1371/journal.pone.0182270 (PMC5536308; doi:10.1371/journal.pone.0182270)
Supplement: S1 Table — Observed/expected number of gender neutral, oogenic, and spermatogenic genes found in the over- and underexpressed proteome (A) and transcriptome (B) of CPEB mutants relative to wild type. Parentheses shows the log2 scaled fold change of observed number relative to expected number (positive value means enrichment and negative value means depletion). Two-tailed Fisher’s exact test P-values are shown at the bottom of each category. (PDF) [file pone.0182270.s014.pdf]

A

Sample	Overexpressed (FC $>$ 1.5 & P-value $<$ 0.05)			Underexpressed (FC $<$ -1.5 & P-value $<$ 0.05)		
	Gender neutral	Oogenic	Spermatogenic	Gender neutral	Oogenic	Spermatogenic
fog-1 [L3]	73/78 (-0.1)<br>3.1×10^{-1}	12/10 (0.26)<br>5.0×10^{-1}	7/7 (0)<br>8.4×10^{-1}	65/62 (0.07)<br>6.2×10^{-1}	8/8 (0)<br>1.0	1/5 (-2.32)<br>4.1×10^{-2}
cpb-2 [L3]	25/27 (-0.11)<br>4.3×10^{-1}	5/3 (0.74)<br>2.0×10^{-1}	5/3 (0.74)<br>1.0×10^{-1}	28/27 (0.05)<br>1.0	1/3 (-1.58)<br>3.6×10^{-1}	2/3 (-0.58)<br>1.0
cpb-3 [L4]	46/44 (0.06)<br>6.7×10^{-1}	3/6 (-1)<br>3.7×10^{-1}	1/4 (-2)<br>1.2×10^{-1}	28/30 (-0.1)<br>6.0×10^{-1}	2/4 (-1)<br>5.8×10^{-1}	4/3 (0.42)<br>3.5×10^{-1}

B

Sample	Overexpressed (FC $>$ 1.5 & BH adjusted P-value $<$ 0.01)			Underexpressed (FC $<$ -1.5 & BH adjusted P-value $<$ 0.01)		
	Gender neutral	Oogenic	Spermatogenic	Gender neutral	Oogenic	Spermatogenic
fog-1 [L3]	524/927 (-0.82)<br>$< 10^{-10}$	121/254 (-1.07)<br>$< 10^{-10}$	221/354 (-0.68)<br>$< 10^{-10}$	1344/965 (0.48)<br>$< 10^{-10}$	232/265 (-0.19)<br>2.1×10^{-2}	242/369 (-0.61)<br>$< 10^{-10}$
cpb-2 [L3]	770/768 (0)<br>9.2×10^{-1}	136/210 (-0.63)<br>1.3×10^{-9}	213/294 (-0.46)<br>1.8×10^{-8}	529/637 (-0.27)<br>5.8×10^{-9}	167/175 (-0.07)<br>5.5×10^{-1}	210/244 (-0.22)<br>1.4×10^{-2}
cpb-3 [L4]	288/494 (-0.78)<br>$< 10^{-10}$	65/135 (-1.05)<br>$< 10^{-10}$	107/189 (-0.82)<br>$< 10^{-10}$	657/610 (0.11)<br>1.0×10^{-2}	381/167 (1.19)<br>$< 10^{-10}$	205/233 (-0.18)<br>3.6×10^{-2}
